# Protein kinase PfPK2 mediated signalling is critical for host erythrocyte invasion by malaria parasite

**DOI:** 10.1371/journal.ppat.1011770

**Published:** 2023-11-21

**Authors:** Rahul Singh Rawat, Ankit Gupta, Neelam Antil, Sonika Bhatnagar, Monika Singh, Akanksha Rawat, T. S. Keshava Prasad, Pushkar Sharma

**Affiliations:** 1 Eukaryotic Gene Expression Laboratory, National Institute of Immunology, New Delhi, India; 2 Institute of Bioinformatics, International Tech Park, Bangalore, India; 3 Amrita School of Biotechnology, Amrita Vishwa Vidyapeetham, Kollam, India; 4 Center for Systems Biology and Molecular Medicine, Yenepoya Research Centre, Yenepoya (Deemed to be University), Mangalore, India; University of Georgia, UNITED STATES

## Abstract

Signalling pathways in malaria parasite remain poorly defined and major reason for this is the lack of understanding of the function of majority of parasite protein kinases and phosphatases in parasite signalling and its biology. In the present study, we have elucidated the function of Protein Kinase 2 (PfPK2), which is known to be indispensable for the survival of human malaria parasite *Plasmodium falciparum*. We demonstrate that it is involved in the invasion of host erythrocytes, which is critical for establishing infection. In addition, PfPK2 may also be involved in the maturation of the parasite post-invasion. PfPK2 regulates the release of microneme proteins like Apical Membrane Antigen 1 (AMA1), which facilitates the formation of Tight Junction between the merozoite and host erythrocyte- a key step in the process of invasion. Comparative phosphoproteomics studies revealed that PfPK2 may be involved in regulation of several key proteins involved in invasion and signalling. Furthermore, PfPK2 regulates the generation of cGMP and the release of calcium in the parasite, which are key second messengers for the process of invasion. These and other studies have shed light on a novel signalling pathway in which PfPK2 acts as an upstream regulator of important cGMP-calcium signalling, which plays an important role in parasite invasion.

## Author summary

Protein kinases, the enzymes involved in phosphorylation of proteins, are critical mediators of cellular signalling. We have unravelled a novel signalling pathway involving protein kinase PfPK2 in human malaria parasite. PfPK2 is indispensable for the parasite development and in the present study we demonstrate that PfPK2 regulates the process of host erythrocyte invasion by the malaria parasite, which is critical for establishing the infection in the host. PfPK2 may regulate this process by targeting proteins involved in signalling and in turn regulate levels of cGMP and calcium levels, which are known to be critical for invasion. In addition to providing insights into novel mechanisms involved in parasite invasion, these studies also highlight the possibility of targeting PfPK2 signalling pathway for blocking malarial infection.

## Introduction

Malaria contributes to almost 600,000 deaths globally in 2021 [1]. While there is a significant reduction in both the number of malaria cases and deaths, the efforts to eradicate this disease have been threatened by the emergence of resistance exhibited by malaria parasite-the most lethal species *Plasmodium falciparum*- against commonly used drugs [2]. Therefore, new therapeutic inventions are needed to keep malaria under control and prevent its rise again. It is the asexual development of the parasite in blood stages that results in malaria pathology [3]. The blood stage development of the parasite starts with the invasion of host erythrocyte by the merozoites. After its entry the parasite forms a parasitophorous vacuole (PV) within which parasite develops from early ring stages to trophozoites, replicates its genome and undergoes asynchronous asexual division in schizont stages. Following segmentation, individual merozoites are generated, which egress after rupture of the host erythrocytes and freshly released merozoites invade new erythrocytes. Erythrocyte invasion is a key step in establishing the infection, therefore, an important process to target for which deep insights into molecular mechanisms are needed [4]. The merozoite has highly specialized organelles- micronemes, rhoptries and dense granules- at its apical end that secrete proteins that serve as ligands for receptors present at the erythrocyte surface. Several receptor-ligand interactions are involved in facilitating the process of invasion, which is a tightly regulated process [5]. After initial contact and attachment, the merozoite reorients to position its apical end for rhoptries to secrete their material. In addition to interacting with receptors on host surface, some microneme proteins like Apical Membrane Antigen-1 (AMA1) are also involved in the formation of Tight Junction with erythrocyte. A family of rhoptry neck proteins (RONs) are also critical for merozoite entry into the erythrocyte [6,7] as some of the RONs interact with microneme secreted AMA1 present on the merozoite surface, which is critical for the formation of Moving or Tight Junction (TJ) between the parasite and the host erythrocyte [7,8]. The actomyosin motor is postulated to provide the force necessary to move the TJ formation towards the basal end, which is critical for parasite entry into erythrocyte [9]. Glideosome complex, which comprises of several proteins, anchors the actomyosin motor to the inner membrane complex and positions it strategically in the merozoite for its function [10,11].

Protein kinases play a critical role during various cellular processes and signalling events regulated by them are important for most cellular functions. ~ 98 putative protein kinases and ~ 30 putative phosphatases are coded in *Plasmodium* genome [12–15]. *Plasmodium falciparum* genome wide knockout studies indicated that several of these kinases are refractory to gene disruption suggesting that they are indispensable for parasite survival [16,17]. Second messengers like calcium, PIPs, cAMP, cGMP *Plasmodium* regulate kinases CDPKs, PKA, PKG etc, which is crucial for diverse parasitic functions [18–23]. For instance, regulation of cAMP dependent protein kinase PKA by cAMP is critical for host erythrocyte invasion [19,24]. PfPKG is involved in egress and invasion of merozoites [23,25] and motility of ookinetes [26]. cGMP and PKG signalling triggers calcium release from parasite stores which is critical for egress as well as invasion facilitated by the release of microneme proteins [23,25,27,28]. Calcium plays a critical role in egress and invasion by virtue of its effector kinases like PfCDPK1 [20,21], PfCDPK5 [29] and phosphatase PfCalcineurin [30].

Protein kinase PfPK2 (Plasmo DB ID: PF3D7_1238900), was previously reported to be activated by calcium/calmodulin *in vitro* [31] and previous studies also demonstrated that it is indispensable for parasite asexual development [16,17]. However, precise function of this protein kinase in the parasite life cycle has remained unknown. Present studies demonstrate that PfPK2 regulates the invasion of host erythrocytes by *P*. *falciparum*. It regulates the secretion of AMA1, which is critical for Tight Junction formation. Phosphoproteomics studies revealed that PfPK2 may target proteins that are implicated in invasion, signalling and glideosome assembly. Furthermore, PfPK2 facilitates the formation of cGMP and the release of calcium from intracellular stores, which are critical for host invasion. These and other observations suggested that it may regulate the process of invasion by regulating parasite signalling networks.

## Results

### Recombinant PfPK2 is not activated by calcium/calmodulin *in vitro*

**Fig 1:**
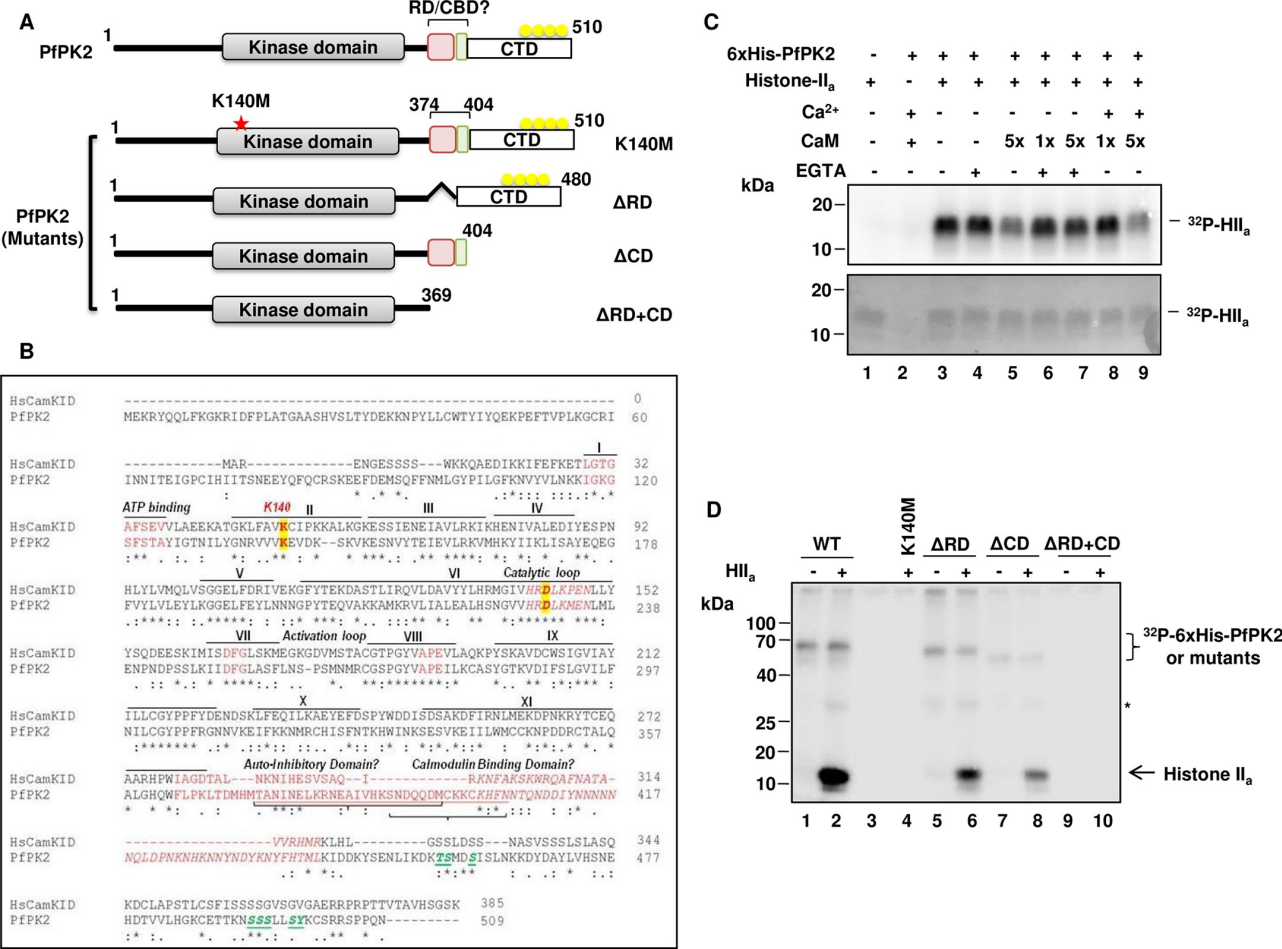
PfPK2 is active *in vitro* independent of calcium/calmodulin. **A.** Schematic indicating domain organization of PfPK2, which includes a putative regulatory domain (RD), a C-terminal domain (CD), which is phosphorylated at several sites in the parasite and a putative calmodulin-binding domain (CBD) predicted by Kato *et al*. (2008). An invariant lysine (K140), which is critical for interaction with ATP in most kinases, is also indicated (red *). Mutants of PfPK2 with K140M mutation or deletion of the RD, CD or RD+CD, which were expressed as recombinant proteins for biochemical analysis, are also indicated. **B.** CLUSTALW alignment of HsCamKID with PfPK2 indicating key motifs, subdomains and critical residues involved in kinase function are highlighted in yellow. The phosphorylation sites identified in the C-terminal domain in the reported phosphoproteomic studies (www.plasmodb.org) are indicated in green. **C**. Purified 6xHis-PfPK2 was used in an *in vitro* kinase assay containing Histone-II_a_ as substrate in a buffer containing [γ^32^P]-ATP in the presence of calcium chloride, 0.1 (x) or 0.5 (5x) μM calmodulin and/or EGTA, which was added to chelate calcium as indicated in the figure. After staining of the SDS-PAGE gel by Coomassie blue (lower panel), phosphorimaging was performed to detect the phosphorylation of the indicated proteins. **D.** Kinase assay was performed using purified 6xHis-PfPK2 or its K140M, ΔRD, ΔCD and ΔRD+CD mutants as described in panel A. *- possibly histone H1 present in Histone IIa preparation. Please see S1B Fig for Coomassie-stained gel used for phosphorimaging.

The analysis of PfPK2 (PlasmoDB ID: PF3D7_1238900) suggested that in addition to a kinase/catalytic domain (a.a. 111 to a.a. 364) it has long N and a C-terminal extensions (Fig 1A). A previous study suggested that PfPK2 shares reasonable sequence homology with CaM Kinase family members like CamKI (~33%) [31,32] but the sequence similarity is highest between kinase domains of human CamKIδ and PfPK2 (~55%) [31]. PfPK2 has a longer N-terminal extension than human CamKI. In addition, CaM binding domain and a regulatory domain were predicted downstream of the kinase domain, which is the case with CamKI [31]. However, a close examination of the sequence in this region suggested a very weak similarity between PfPK2 and CamKIδ in this region (Fig 1A and 1B). In addition, PfPK2 also has a long C-terminal extension, which is phosphorylated at several sites in the parasite as indicated by previous phosphoproteomics studies [33] (www.plasmodb.org). Previously, calcium/calmodulin (CaM) was reported to activate PfPK2 *in vitro* [31].

Recombinant PfPK2 and its mutants were expressed as a 6xHis-tagged protein in *E*. *coli* for biochemical characterization (S1A Fig). A kinase assay was performed in which Histone II_a_ was used as a phosphoacceptor substrate. Recombinant PfPK2 phosphorylated histone II_a_ (Fig 1C). However, there was no increase in its activity despite addition of excess calcium/calmodulin. In contrast, there was a slight decrease in histone phosphorylation when calcium/calmodulin was added to the reaction mix. These data suggested that PfPK2 may not be activated by calcium/calmodulin, at least *in vitro*.

A lysine at 140 position (K140) (Fig 1B), which is complementary to an invariant lysine present in subdomain II of most PKs and is critical for their activity [34], was mutated to M (K140M). K140M mutant did not exhibit any autophosphorylation or Histone phosphorylation (Fig 1D), which suggested that this residue is critical for PfPK2 activity. Next, we analyzed the role of the C-terminal domain (CD) and putative regulatory domain (RD) in PfPK2 regulation. For this purpose, deletion mutants lacking these domains were generated and recombinant proteins were used for activity assays (S1A and S1B Fig). The deletion of RD or CD caused a decrease in the phosphorylation of Histone II_a_ by PfPK2 (Fig 1D). A marked decrease in autophosphorylation was also observed in the case of ΔRD, which was even more significant in the case of the ΔCD mutant. The deletion of both ΔRD and ΔCD resulted in almost complete loss of PfPK2 activity. Collectively, these data suggested that at least *in vitro* these domains are critical for PfPK2 activity. It is possible that the phosphorylation of CD, which is also phosphorylated at several sites in the parasite [33] (www.plasmodb.org), may have a role in its parasitic functions like interaction with other proteins.

### Role of PfPK2 in the development of *P*. *falciparum*

#### Conditional knockout of PfPK2 in *P*. *falciparum*

**Fig 2:**
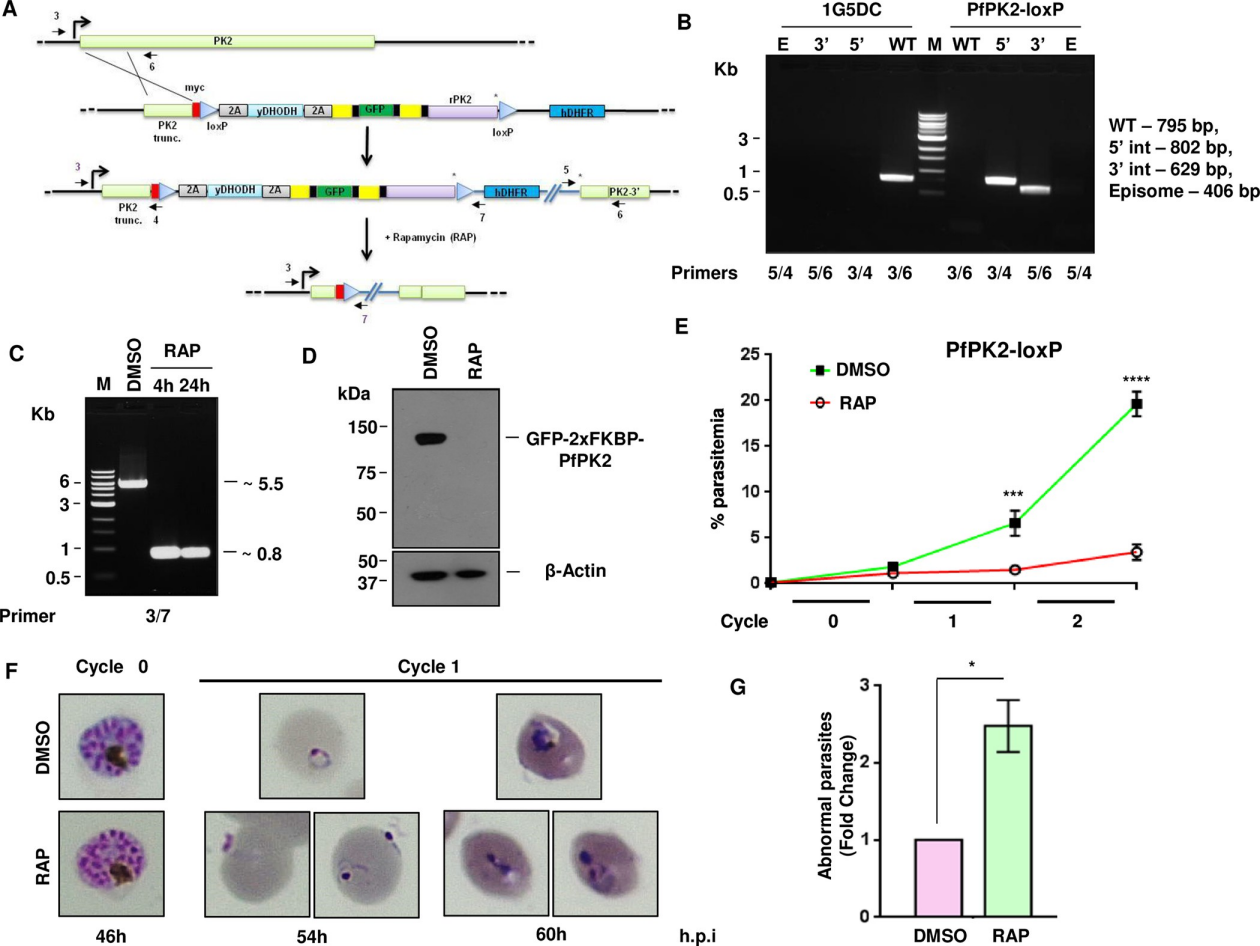
PfPK2 is critical for asexual phase life cycle of *P*. *falciparum*. **A.** Schematic representation of the strategy used to modify the *PfPK2* locus through single-crossover homologous recombination and selection-linked integration (SLI). A 330bp region homologous to the 5’-end of *PfPK2* gene was cloned in pSLI-N-sandwich-loxP vector. In addition, recodonized PfPK2 (rPK2) was cloned in frame with GFP (green). Yellow- FKBP, black- linker, triangles -loxP, *-stop codon, arrows- location of annealing site of PCR primers used for genotyping and assessing excision, which was achieved after rapamycin addition. **B.** Genotyping of a clone obtained after limited dilution (PfPK2-loxP). PCR amplification was carried out for both the unmodified and modified loci, using primers targeting the indicated sites in Panel A. PCR products of expected size (indicated in the figure) were amplified confirming 5’- and 3’-integration in the transgenic PfPK2-loxP parasite. While amplification for the unmodified wild type (WT) locus was observed in 1G5DC parasites, it was absent in PfPK2-loxP parasites. The amplification for episome (E) was not detectable under the same conditions. The amplicons were sequenced to further confirm the integration at the desired locus. **C.** PfPK2-loxP parasites were treated with DMSO or 200 nM RAP for 4 or 24h. Subsequently, genomic DNA was isolated ~44 hpi and PCR was performed using indicated primer sets (panel A). A ~0.8kb product was obtained upon RAP treatment indicating successful excision. **D.** Western blot examination of PfPK2-loxP parasite lysates treated with DMSO and RAP and using anti-GFP antibody revealed that the RAP-treatment resulted in effective depletion of GFP-PfPK2. As a loading control, a β-actin antibody was also used to probe the blots. **E.** PfPK2-loxP parasites were synchronized and ring stage parasites were used for setting up growth rate assay in the presence of DMSO or RAP. After 6h, RAP was washed and parasite growth was assessed after each cycle by performing flow cytometry (SEM ± SE, n = 3, ANOVA, ***, p<0.001, **** P<0.0001; ns—not significant). **F.** PfPK2-loxP parasites were treated with DMSO or RAP as described in panel A and Giemsa-stained blood smears at indicated time post invasion (h.p.i) were examined. PfPK2-depleted parasites showed significant parasites attached to the erythrocytes were suggestive of failed invasion. A few parasites that entered erythrocytes exhibited abnormal morphology and/or were pyknotic. **G.** The number of abnormal/pyknotic parasites -indicated in panel F-were counted after RAP addition (72 h.p.i, ~40 parasites were counted for each replicate), which revealed a significant increase in number of abnormal/pyknotic RAP-treated parasites when compared to DMSO-treated controls (SEM ± SE, n = 3, * P<0.05, paired t test).

Previous attempts to knockout PfPK2 from *P*. *falciparum* have been unsuccessful suggesting that this kinase may be indispensable for blood stage development of the parasite [16]. However, its role in *P*. *falciparum* remained undefined. We used a rapamycin-inducible dimerizable Cre recombinase (diCre) based strategy for conditional knockout of PfPK2 and used 1G5DC strain [35], which expresses FKBP and FBP that are independently fused to two halves of the Cre recombinase and dimerize upon addition of rapamycin (RAP) resulting in active Cre enzyme, which can then facilitate the excision of the genetic sequence flanked by two loxP sites. Using Selection Linked Integration (SLI) [36] loxP sites were introduced in the PfPK2 locus (Fig 2A) and a recodonized copy of PfPK2 was introduced downstream of GFP. Parasites obtained after drug (WR99210 and DSM1) selection were subjected to limiting dilution cloning. Correct integration of the targeting plasmid at the desired locus was confirmed by PCR as amplicons of expected size were obtained from a clone that lacked wild type PfPK2 (Fig 2B). The addition of rapamycin (RAP) to PfPK2-loxP parasites for 4h or 24h at ring stages resulted in efficient excision of the floxed sequence at the schizont stage of the same cycle (Fig 2C). Successful expression of GFP-PfPK2 was indicated by Western blotting (Fig 2D) as well as by IFA (S3A Fig), which was undetectable after rapamycin treatment suggesting efficient depletion of this kinase from the parasite (Figs 2D and S3B). Immunofluoroscence assays (IFA) indicated that PfPK2 localized to punctate vesicular structures often present apically near the micronemes but did not exhibit any significant co-localization with microneme (AMA1) or rhoptry (RhopH3) proteins (S3A Fig).

### PfPK2 is important for erythrocyte invasion and asexual development of *P*. *falciparum*

To investigate the role of PfPK2 in parasite development, synchronized ring stage cultures of 1G5DC (control) or PfPK2-loxP lines were treated with RAP to promote the excision of PfPK2. PfPK2-loxP parasites did not exhibit much change in parasitemia in the cycle of RAP-treatment (cycle 0). However, the parasite growth was significantly reduced at the onset of the next cycle and very few parasites were observed subsequently (Fig 2E). There was almost no apparent change in number of merozoites formed upon RAP-treatment during cycle 0 (S4B Fig) suggesting parasite replication was unaffected. Importantly, parental 1G5DC strain was unaltered by RAP addition (S2 Fig).

A close examination of Giemsa- stained blood smears of parasite cultures at the beginning of cycle 1 revealed a very small number of ring-infected erythrocytes upon PfPK2 depletion, which was indicative of possible defects in egress and/or invasion (discussed below in detail). A very small number of parasites that managed to invade exhibited stunted abnormal morphology and/or were pyknotic and failed to mature to trophozoites (Fig 2F and 2G).

**Fig 3:**
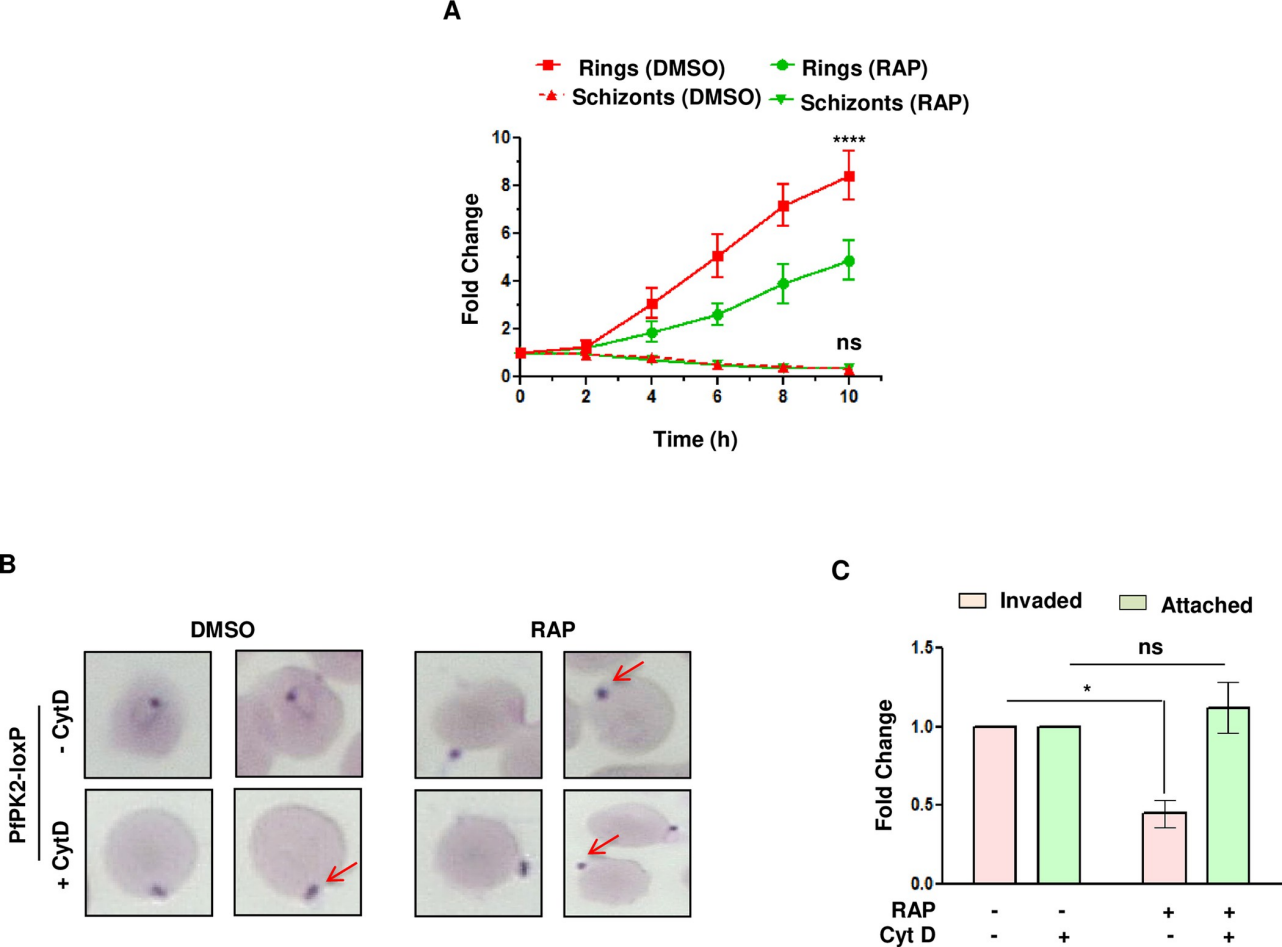
PfPK2 regulates the process of host erythrocyte invasion. **A.** PfPK2-loxP schizonts treated with DMSO or RAP were incubated with fresh erythrocytes. After indicated time, samples were collected and the number of schizonts and rings were counted from Giemsa-stained thin blood smears and fold change in schizont or ring-infected erythrocytes was determined. RAP-treated parasites showed a significant decrease in the number of rings whereas schizont numbers were largely unaltered and almost no schizonts were observed at the end of the assay (SEM ± SE, n = 6, ANOVA, **** P<0.0001; ns—not significant). **B and C.** DMSO or RAP treated PfPK2-loxP parasites were used for erythrocyte attachment assays which were performed in the presence or absence of cytochalasin D. The number of merozoites attached to erythrocytes were counted from Giemsa-stained thin blood smears (B) and fold change in % attachment (C) with respect to DMSO treated parasites was determined (SEM ± SE, n = 3, ANOVA, * P<0.05; ns -not significant).

The possibility of defects in egress and/or invasion upon PfPK2 depletion was investigated in detail. For this purpose, assays were performed using DMSO or RAP-treated mature schizonts (cycle 0, ~40–44 hpi, treatment performed at the ring stage), which were incubated with fresh erythrocytes. The schizont number consistently reduced with almost no residual schizonts left after a few hours, which suggested that the egress was not impaired in PfPK2-depleted parasites (Fig 3A). In contrast, there was a marked decrease in the number of fresh rings formed in RAP-treated parasites (Fig 3A), which was indicative of impaired invasion. A parasite line was generated in PfPK2-loxP background in which a WT copy of HA-tagged PfPK2 was expressed episomally to complement the PfPK2 function (cHA-PfPK2-loxP) as RAP does not influence the expression of HA-PfPK2 (S4A Fig). Importantly, there was no significant difference in invasion as well as post-invasion defects reported above in the case of cPfPK2-loxP parasites (S4C and S4D Fig) confirming the involvement of PfPK2 in invasion and in these processes. PfPK2 was not expressed in gametocytes (S5 Fig), which possibly rules out its involvement in conversion of parasites to sexual forms.

**Fig 4:**
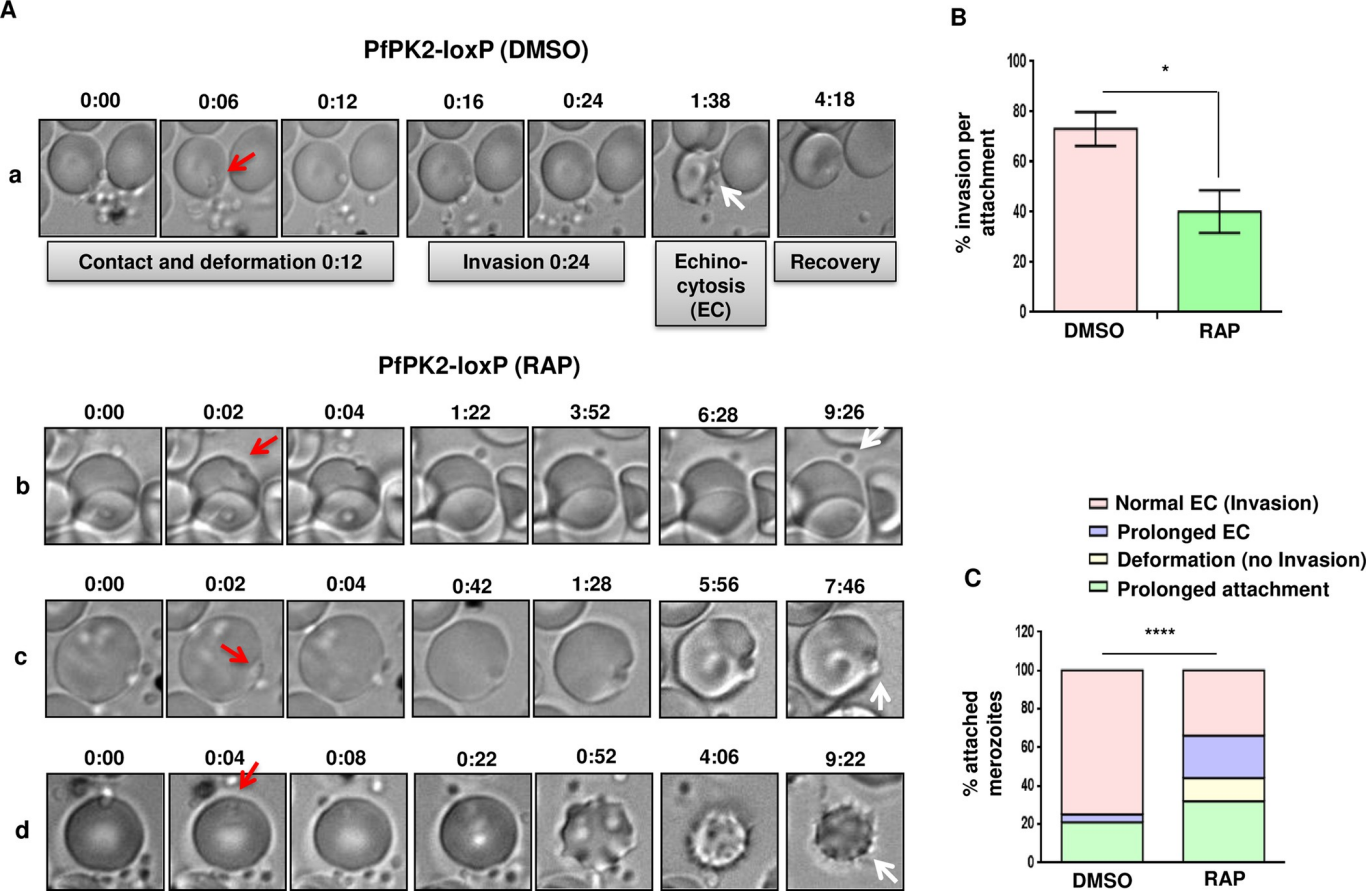
PfPK2 depletion impairs the entry of merozoites in host erythrocytes. **A.** Schizonts isolated from PfPK2-loxP parasites cultured in the presence or absence (DMSO) of RAP for one cycle and post-egress merozoites were used for live microscopy (S1–S4 Video). Selected still images from videos are used to illustrate various stages of invasion. DMSO-treated PfPK2-loxP merozoites successfully deform the erythrocyte membrane (red arrow), invade, and trigger echinocytosis (white arrow) followed by recovery of the infected erythrocyte (a, video S1). Prolonged attachment upon RAP-treatment and unsuccessful invasion was observed (b, video S2). Several RAP-treated parasites that exhibited prolonged attachment (red arrow) were also able to deform the erythrocyte membrane (c, S3 Video) and stimulate echinocytosis (EC) which was for much longer duration (d, white arrow, S4 Video). **B.** % invasion per attachment was determined and data represent results from four independent pooled experiments described in panel A (mean ± SE, n  =  4, *, P < 0.05, paired t test). **C.** Various outcomes during erythrocyte invasion indicated in Panel A were quantitated for merozoites that formed an initial attachment using data from four different experiments for each set of conditions [****, P < 0.0001, chi-square test].

Further investigations were performed to determine which stage of host erythrocyte invasion, which is a complex multi-step process [37,38], is regulated by PfPK2. Upon release after the rupture of erythrocytes, merozoites first attach to the surface of fresh erythrocytes and several ligand-receptor interactions are involved in this process. The role of PfPK2 in attachment was probed by using cytochalasin D (Cyt D), which prevents invasion or entry but does not affect attachment [39]. There was no significant difference in the number of DMSO or RAP-treated PfPK2-loxP parasites attached to erythrocytes (Fig 3B and 3C), which suggested that PfPK2 may regulate an event post-attachment. To better understand defects in the invasion following PfPK2 depletion live cell imaging was performed. Typically, merozoite invasion starts with the attachment of the merozoite to the host erythrocyte resulting in the deformation of the erythrocyte membrane [38]. Subsequently, the merozoite reorients its apical end on to the erythrocyte surface followed by its penetration into the erythrocyte. Once internalized, the infected erythrocyte undergoes echinocytosis for a short period followed by its recovery. Subsequently, erythrocyte membrane seals and the parasite attains ring like morphology (Fig 4Aa, S1 Video) [38,40]. Video microscopy revealed significantly lesser RAP-treated merozoites invading the erythrocytes in comparison to DMSO-treated counterparts (Fig 4B, S1–S4 Video,). Upon RAP-treatment most parasites remained attached to the erythrocyte surface and exhibited re-orientation with apical end positioned to enter the erythrocyte (Fig 4Ab, Fig 4Ac, S2 Video,). The attachment was prolonged, but parasites failed to penetrate the erythrocyte. The attached merozoites were able to deform the host erythrocyte membrane and caused echinocytosis for a significantly longer duration in RAP- treated parasite (Fig 4Ac, Fig 4Ad, S3–S4 Video). These observations suggested that PfPK2 depletion may not influence attachment, but it prevents entry possibly at a late step after interaction between the merozoite and the erythrocyte surface, which was supported by the fact that attached parasites caused deformation as well as echinocytosis. Collectively, these observations suggested a role of PfPK2 in later-steps of invasion like formation of Tight Junction and possibly subsequent sealing of the erythrocyte.

### PfPK2 regulates the discharge and shedding of AMA1 from the parasite

**Fig 5:**
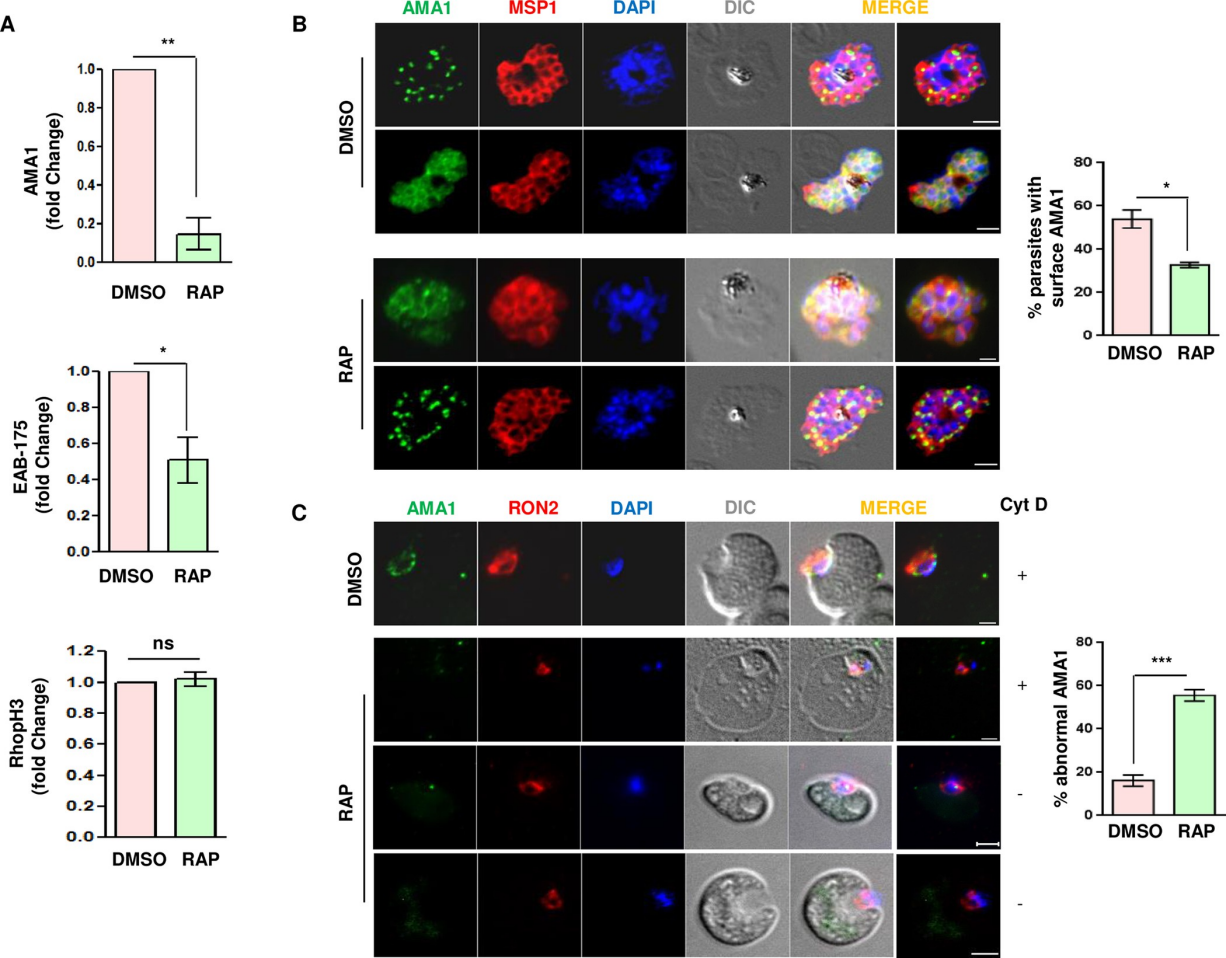
PfPK2 regulates microneme release. **A.** DMSO or RAP treated PfPK2-loxP schizonts were cultured for one cycle and the release of AMA1, EBA175 and RhopH3 from parasites was determined by performing Western blotting on culture supernatant using specific antibodies (S6 Fig). The secretion of these proteins in the supernatant was quantitated by densitometry of the Western blot, and the fold change in secretion for RAP-treated parasites with respect to DMSO-treated parasites was determined (AMA1, mean ± SEM, n  =  4, **, P < 0.01; EBA175, mean ± SEM, n  =  4, *, P < 0.05; RhopH3, mean ± SEM, n  =  4, ns, P > 0.05, ns- not significant; paired t test). Western blots were also performed on total parasite protein lysates, which were used for normalization of secreted proteins (S6 Fig). **B.** PfPK2-loxP parasites were treated with DMSO or RAP. Subsequently, schizonts were treated with E64 to prevent egress and IFA was performed to detect AMA1 and MSP1. DAPI was used to stain the nucleus, DIC provides the bright field image of the parasite. AMA1 localizes to microneme in schizonts stage (DMSO, upper panel) but during egress it comes to the merozoite surface in a significant number of parasites (DMSO, lower panel). In RAP-treated parasite while some parasites exhibited AMA1 on the surface (RAP, upper panel) in a signficant number of parasites it was retained in the micronemes in most parasites (RAP, lower panel), which was confirmed by counting parasites (Right panel) with AMA1 staining on the surface (mean ± SEM, n = 3, * p<0.05, paired t test). **C.** The presence of AMA1 at the Tight Junction in DMSO or RAP-treated PfPK2-loxP parasites was determined by performing IFA for AMA1 and RON2. In the case of DMSO-treated parasites, AMA1 localized proximal to RON2 whereas it was either not detected or it was significantly reduced in RAP-treated parasites. *Right panel*, parasites exhibiting abnormal or no AMA1 staining were quantitated (SEM ± SE, n = 3, ANOVA, *** P<0.001).

As mentioned above, discharge of ligands from secretory organelles like micronemes and rhoptries is critical for invasion [5,37]. Therefore, we assessed the release of rhoptry and microneme proteins in parasite culture supernatants. There was almost no difference in the release of rhoptry protein RhopH3 upon RAP treatment (Figs 5A and S6C) excluding the possibility of involvement of PfPK2 in rhoptry release. Interestingly, the shedding of microneme protein AMA1—which is involved in Tight Junction (TJ) formation [7,41–43]–was dramatically reduced upon PfPK2 depletion (Figs 5A and S6A). The release of another microneme protein EBA-175, which interacts with glycophorin A on the surface of the erythrocytes [44], was also reduced (Figs 5A and S6B) but its localization to the microneme was unaltered (S7 Fig). These data suggested that PfPK2 may regulate microneme release. However, there was no difference in the attachment of parasites (Fig 3C), which is regulated by adhesins like EBA1-75. Therefore, we focussed on a possible role of PfPK2 in the release of AMA1, which is involved in late invasion events like Tight Junction formation and PfPK2 plays a role at this late stage of invasion (Fig 4). AMA-1 is first secreted to the merozoite surface from micronemes and subsequently shed or released by the action of proteases like SUB2 and rhomboid proteases [45]. AMA1 was found in micronemes as indicated by a puncta at the apical end in both DMSO and RAP- treated parasites but a significant population was found on merozoite surface in the former. In contrast, there was a decrease in parasites with AMA-1 on their surface upon PfPK2 depletion and it was confined mainly to the micronemes (Fig 5B). Therefore, it appears PfPK2 may regulate the secretion of AMA-1 on to the merozoite surface and in its shedding from merozoite surface.

AMA1 plays a role in the formation of Tight-Junction (TJ) *via* its association with rhoptry neck proteins (RONs) that are released by the rhoptries "Just-in-Time" on to erythrocyte surface [46]. IFAs were performed to localize AMA1 along with RON2 in "invading" merozoites. As reported previously [46–48], AMA1 either forms a ring around the surface and/or was concentrated at the points of contact between the merozoite and the erythrocyte membrane along with RON2 (Fig 5C). RAP-treatment caused a dramatic decrease in AMA1 staining, which was either not observed or at best was seen as a small puncta that did not co-localize with RON2 (Fig 5C). These data were consistent with the fact that AMA1 secretion was impaired upon PfPK2 depletion (Fig 5A). Given that AMA1 is critical for TJ formation [43], it is reasonable to state that PfPK2 may contribute to this process.

### Identification of PfPK2 targets by quantitative phosphoproteomics

**Fig 6:**
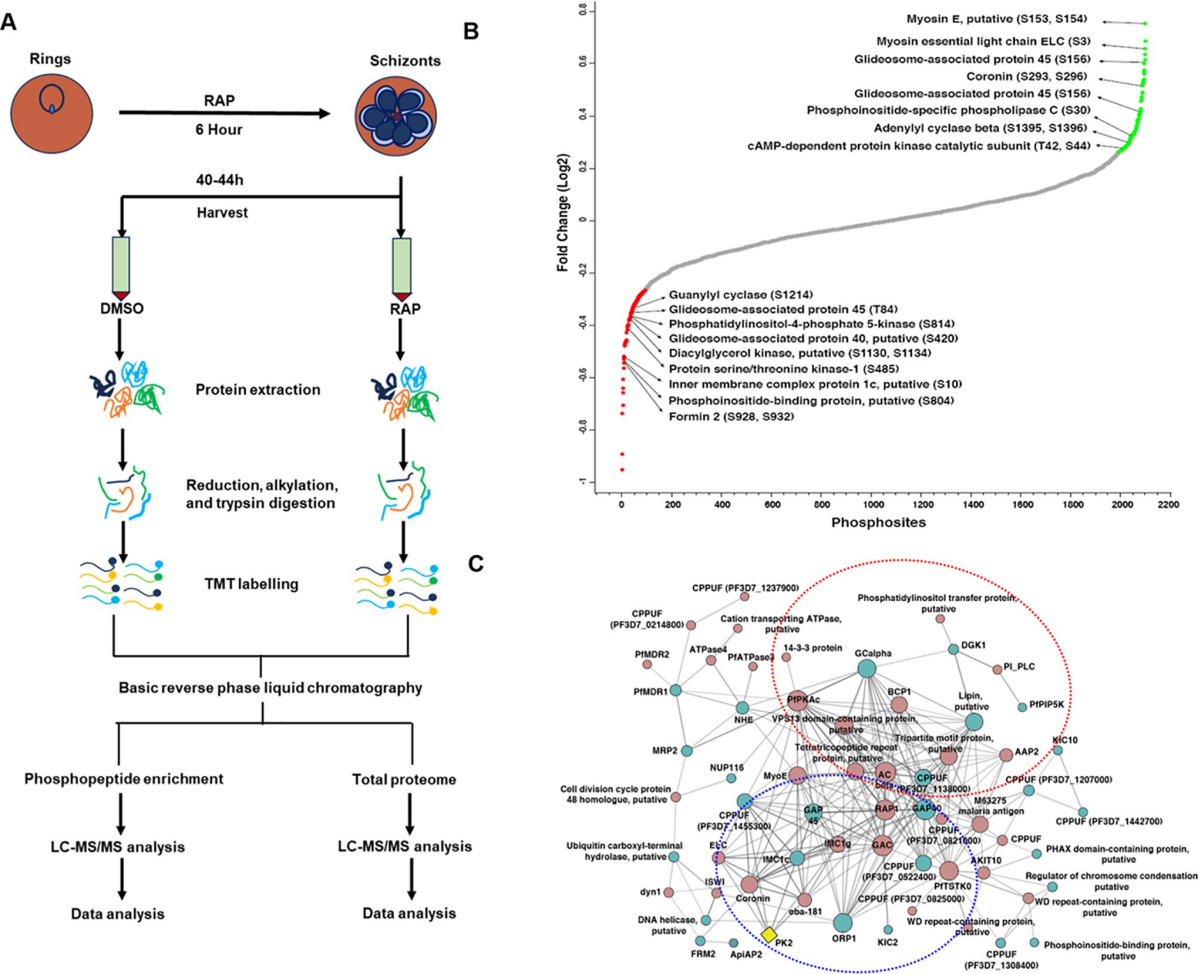
Identification of PfPK2 targets in the parasite by comparative phosphoproteomics. **A.** PfPK2-loxP parasites were synchronized to obtain rings, which were treated with DMSO (control) or RAP and parasites were harvested at schizont stage to perform comparative phosphoproteomic and proteomic analyses as indicated in the schematic (See Methods for details). **B.** The phosphorylation fold-change ratios of identified phosphopeptides were normalized to total protein abundance fold-change. The fold-change ratios for all phosphopeptides from various replicates are provided in S1 Dataset, 1.1 The S-curve for the normalized data from three biological replicates is provided, some of the significantly altered phosphorylation sites belonging to key proteins (S1 Dataset, 1.2, 1.3) are highlighted. **C.** Protein-protein interactions were predicted between differentially phosphorylated proteins using the STRING resource. The analysis exhibited high confidence interactions between the candidate proteins (S1 Dataset, 1.5). Two major protein-protein interaction clusters involving proteins implicated in signalling and glideosome/invasion (Table 1) are encircled in red and blue, respectively. Red and green nodes illustrate hyperphosphorylated and hypophosphorylated proteins, respectively. The thickness of the circles represents the number of interactions with the representative node. PfPK2 was manually included for the ease of understanding its putative direct/indirect interacting partners and associated biological processes. The term "CPPUF" refers to a Conserved *Plasmodium* Protein with an Unknown Function.

In order to elucidate the mechanism via which PfPK2 may regulate parasite invasion, it was important to identify the targets of PfPK2 and unravel PfPK2-dependent signalling pathways. Therefore, quantitative phosphoproteomics was used to compare the phosphoproteome of PfPK2-loxP depleted parasites in the presence or absence of RAP (Fig 6A). PfPK2 expression was maximal in late schizonts/merozoites during asexual blood stage development, which correlates well with defects in invasion (Fig 4). Therefore, it was pertinent to use late schizonts for phosphoproteomics. The lysates of RAP or DMSO-treated PfPK2-loxP parasites were harvested at the late schizont stage, and phosphoproteomic analysis was performed on the prepared lysates. Phosphoproteomics identified 2894 phosphopeptides in the PfPK2-loxP parasites and comparison of the phoshoproteome of DMSO and RAP-treated parasites indicated that several sites on these peptides exhibited differential phosphorylation upon PfPK2 depletion achieved by RAP treatment (Fig 6B). A total of 104 phosphosites on 82 proteins exhibited significant reduction (p≤0.05, fold change ≥1.2) in phosphorylation upon PfPK2 depletion (Fig 6B, S1 Dataset). It was interesting to note that several proteins with well-defined or proposed functions in invasion exhibited significant changes in phosphorylation, including proteins involved in signalling pathways and actomyosin motor function (Fig 6B, Table 1). Strikingly, several key proteins were also hyperphosphorylated in PfPK2-depleted parasites, which may be due to direct or indirect effects exerted by PfPK2 on a protein phosphatase or by negatively regulating other protein kinases. For instance, protein phosphatase PPM2 was found to be hyperphosphorylated upon PfPK2 depletion (Fig 6B, S1 Dataset, 1.2), which may potentially contribute to the phosphorylation status of some of the aberrantly phosphorylated proteins.

**Table 1:** Key putative parasitic targets of PfPK2 that are implicated in invasion and signalling.

Phosphorylated proteins	PlasmoDB ID	Phosphosites
Signalling		
Adenylyl cyclase β Protein phosphatase PPM2 cAMP-dependent protein kinase catalytic subunit PI-Phospholipase C	PF3D7_0802600 PF3D7_1138500 PF3D7_0934800 PF3D7_1013500	S1395, S1396 S139, T142 T42, S44 S30
Phosphatidylinositol-4-phosphate 5-kinase Diacylglycerol kinase, putative Guanylyl cyclase α	PF3D7_0110600 PF3D7_0930500 PF3D7_1138400	S814 S1130, S1134 S1214
Invasion/Egress		
Erythrocyte binding antigen-181 Rhoptry-associated protein 1 Merozoite surface protein 2	PF3D7_0102500 PF3D7_1410400 PF3D7_0206800	S1299 S140, S169, S294 S214
Rhoptry-associated protein 1	PF3D7_1410400	S127
Glideosome/IMC		
Coronin Inner membrane complex protein 1c, putative Inner membrane complex protein 1g, putative Myosin E, putative Myosin essential light chain (ELC) Glideosome-associated protein (GAP) 45 Glideosome-associated connector (GAC)	PF3D7_1251200 PF3D7_1003600 PF3D7_0525800 PF3D7_0613900 PF3D7_1017500 PF3D7_1222700 PF3D7_1361800	S293, S296 S266, S267 S277 S153, S154 S3 S156 T564, S566
Glideosome-associated protein 40, putative Inner membrane complex protein 1c, putative Glideosome-associated protein 45	PF3D7_0515700 PF3D7_1003600 PF3D7_1222700	S420 S10 T84

A subset of significantly altered hyper- and hypo-phosphorylated sites detected upon PfPK2 depletion (S1 Dataset) which may be relevant for PfPK2 function in the process of invasion are indicated in pink and green boxes, respectively.

The above-mentioned family of proteins were of special interest as they are likely to be relevant for the function of PfPK2 in host erythrocyte invasion. Glideosome associated proteins PfGAP45 and PfGAP40 exhibited altered phosphorylation upon PfPK2 depletion. These proteins are important for anchoring the motor and thereby regulate actomyosin motor activity [11,49], which is essential for erythrocyte invasion [50]. Previously, phosphorylation of PfGAP45 was reported by several studies which is considered to be important for invasion [51,52].

The catalytic subunit of cAMP-dependent protein kinase PfPKA exhibited hyperphosphorylation upon PfPK2 depletion. Strikingly, PfPKAc hyperphosphorylation was observed at T42 and S44 (Table 1, S1 Dataset, 1.2) which coincides with the ATP binding region that comprises of a GxGxxG motif. The phosphorylation of these sites may have a bearing on interaction of ATP with the kinase and inhibit the activity of PfPKA [53,54]. In addition, adenylyl cyclase (ACβ) was also found to be hyper phosphorylated. These observations hinted at possible involvement of the cAMP signalling module downstream of PfPK2 and altered phosphorylation of these proteins can have a bearing on cAMP signalling and PfPKA activity. Interestingly, PfPKA has been shown to play a role in erythrocyte invasion by the parasite by several groups [19,24,55].

Strikingly, PfPK2 depletion prevented phosphorylation of gunaylyl cyclase PfGCα (Fig 6B, Table 1), which is involved in generating second messenger cGMP. cGMP signalling regulates host erythrocyte invasion as well as egress [23,25,27,56]. Several proteins implicated in phosphoinositide (PIP) metabolism and or downstream effectors of PIP signalling were aberrantly phosphorylated upon PfPK2 depletion: A PI4P-5-Kinase, which is involved in generation of PI(4,5)P2, was hypophosphorylated at S814 (Fig 6B, Table 1, S1 Dataset, 1.3).

PI4,5P2 can regulate signalling via downstream effector proteins and/or serve as substrate for PI-PLC. Interestingly, PfPLC phosphorylation at S30 increased in PfPK2-depleted parasites (Fig 6B, Table 1, S1 Dataset, 1.2). PLC hydrolyses PI(4,5)P2 to yield I(1,4,5P3) (IP3) and diacylglycerol (DAG), which are potent second messengers. IP3 regulates calcium release from the ER in the parasite [57] like other organisms but IP3 receptor has not been identified, although a transmembrane protein has been implicated in this process [58]. PLC-mediated calcium release has been implicated in the process of invasion [57]. DAG is substrate for DAG-kinase, which converts it to phosphatidic acid (PA). Recently, DAGK inhibition was shown to block gliding motility which is important for invasion [9]. Therefore, it was interesting to note that the phosphorylation at S1130 and S1134 was reduced when PfPK2 was depleted (Fig 6B, Table 1, S1 Dataset, 1.3). Given that these pathways are involved in invasion, it is reasonable to suggest that PfPK2 may regulate invasion by possibly regulating cGMP and/or phosphoinositide signalling in the parasite.

Protein-protein interaction (PPI) networks among differentially phosphorylated proteins (Fig 6C) were predicted using the STRING database, which uses information from reported experimental results, co-expression profiles etc [59]. Consistent with observations made above, two major PPI- modules that related to PfPK2 function in invasion emerged which comprised of: a. proteins related to invasion like surface, rhoptry and microneme proteins (e.g., RAP1, EBA181) or gideosome/actomyosin motor related proteins (GAP45, GAC, GAP40, MyoE); b. signalling proteins like GCα, PKAc, ACβ, PLC (Fig 6C). These putative PPI-networks provided first indication of the molecular processes possibly regulated by PfPK2 during the process of invasion.

### PfPK2 regulates cGMP levels and calcium release in the parasite

**Fig 7:**
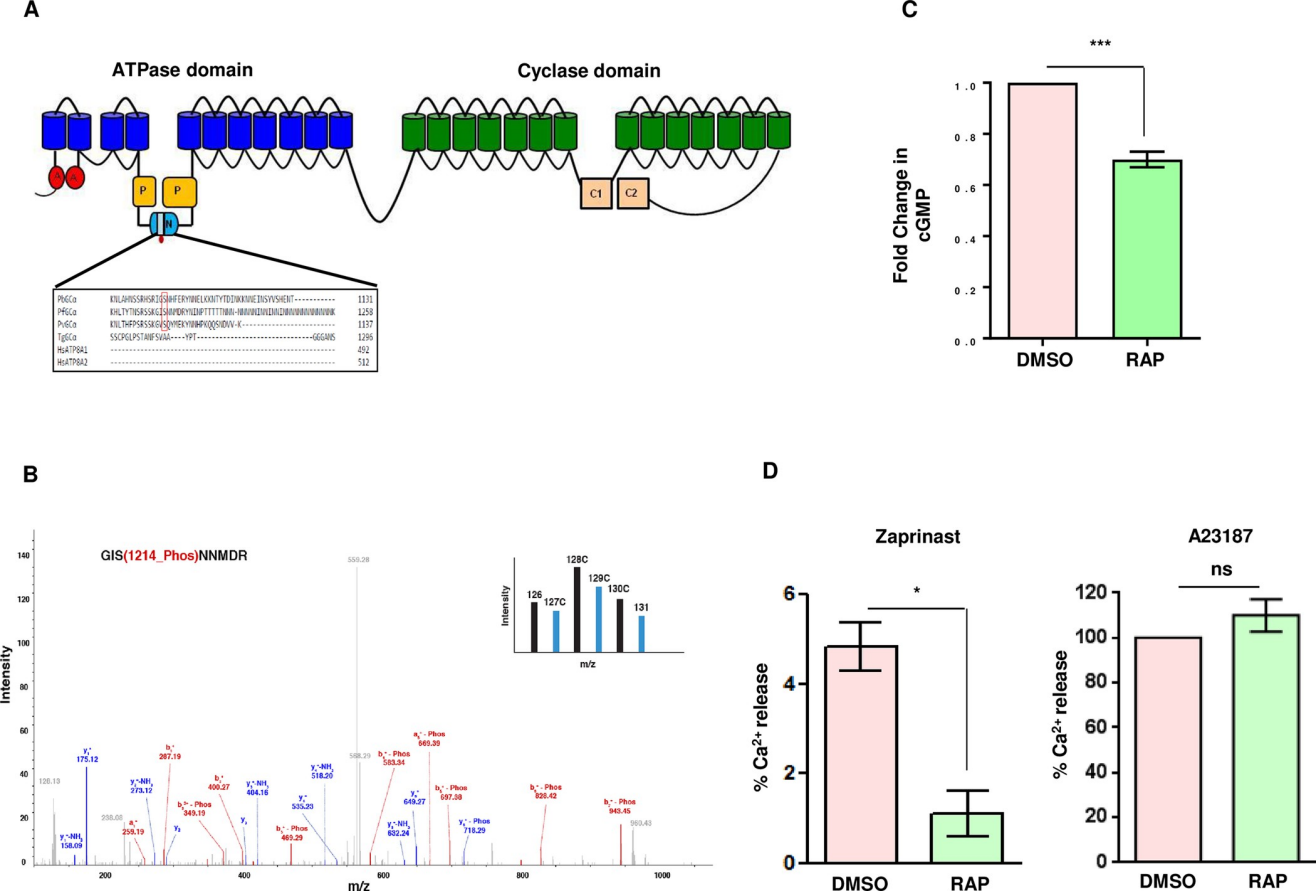
PfPK2 regulates calcium release and cGMP levels in the parasite. **A.** Schematic illustrating the domain architecture of PfGCα, which has an ATPase domain fused to the guanylyl cyclase, various subdomains are indicated [27]. The Nucleotide binding domain (N), which splits the phosphorylation (P) domain, has a long insert and PfPK2-regulated phosphorylation site S1214 resides in an insert. Sequence alignment of GCα from *P*. *falciparum*, *P*. *berghei and Toxoplasma gondii* and human ATPase ATP8A1/2 indicates that this site is conserved only in *Plasmodium* spp (S8 Fig). **B.** The phosphorylation of S1214 of PfGCα was reduced upon PfPK2 depletion. MS/MS spectra is provided for the corresponding phosphopeptide. TMT channel indicated in the inset represents independent biological replicates of PfPK2 DMSO (black) or RAP (cyan) parasites and wild type samples. **C.** DMSO or RAP-treated PfPK2-loxP schizonts were lysed and an ELISA-based assay kit was used to quantitate intracellular cGMP. The data provides fold change in cGMP upon RAP-treatment (mean ± SEM, n  =  5, ***, P < 0.001, paired t test). **D.** Fluorimetry was used to assess calcium mobilization, which was determined by using Fluo-4-loaded mature DMSO- and RAP-treated PfPK2-loxP schizonts that were treated with zaprinast (left panel) or A23187 (right panel). The signals were normalised with respect to DMSO (0%) and A23187 (100%). The data presented are mean of four independent biological replicates that were done in duplicate (mean ± SEM, n  =  4, *, P < 0.05, ns, P > 0.05, ns- not significant, paired t test).

As described above, phosphoproteomic studies indicated that PfPK2 depletion altered the phosphorylation of several signalling proteins that includes guanylyl cyclase GCα coded by gene PF3D7_0381400 (Figs 6B, 7A and 7B and Table 1) which synthesizes cGMP in the parasite [27]. In the case of *Plasmodium spp*., GCs are assembled in a peculiar configuration in which the cyclase domain comprising of the two catalytic domain is fused to a P4-ATPase domain [18]. The nucleotide binding domain (N-domain), which is important for ATP binding, has large inserts in the case of PfGCα [27]. Interestingly, phosphorylation site S1214 which is regulated by PfPK2 (Figs 6B and 7B) resides in this insert. This phosphosite is conserved only in GCα from *Plasmodium* spp (Fig 7A) and is absent from human ATPase8A1/2.

To explore if PfPK2 depletion has an impact on cGMP synthesis, levels of this cyclic nucleotide were measured in the parasite. A phosphodiesterase (PDE) inhibitor zaparinast, which prevents cGMP degradation, was used to maintain cGMP levels for detection. Strikingly, PfPK2-depletion caused a marked decrease in cGMP formation upon PfPK2 depletion indicating its role in the synthesis of this second messenger (Fig 7C).

Given that calcium release in *P*. *falciparum* is regulated by cGMP [27], it was pertinent to assess the role of PfPK2 in calcium release. For this purpose, parasites were loaded with cell permeable calcium-sensitive fluorophore Fluo-4-AM to measure free calcium in the parasite as described previously [27,60]. Phosphodiesterase inhibitor zaprinast—which causes sustained calcium release from intracellular stores [27,61], was used to treat PfPK2-loxP parasites. One set of parasites was also treated with calcium ionophore A23187 to estimate the amount of total stored calcium. RAP- treatment of PfPK2-loxP parasites caused a significant decrease in calcium release as indicated by the data from four independent biological replicates and almost no change in A23187 induced calcium release was observed. These data suggested that PfPK2 is involved in the release of calcium from intraparasitic stores (Fig 7D).

**Fig 8:**
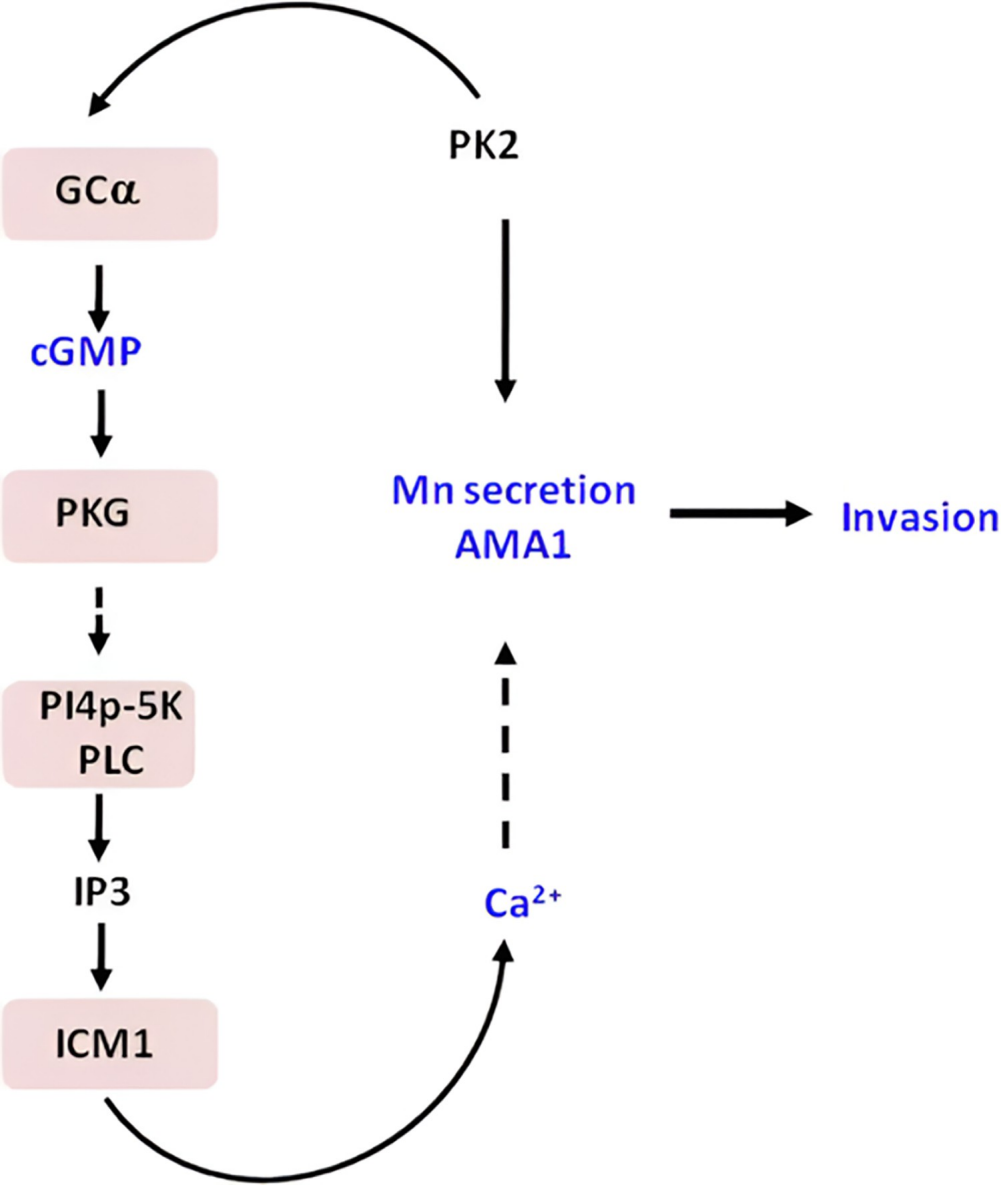
A novel PfPK2- dependent signalling pathway was identified in which PfPK2 may promote cGMP production (Fig 7C) by regulating GCα (Figs 6Β and 7Α), which in turn may facilitate the release of calcium (Fig 7D) from intraparasitic stores and activate PfPKG, which is known to trigger calcium release in the parasite [18,26]. Calcium, which is critical for the invasion, may also facilitate the release of microneme proteins like AMA1 (Fig 5), which is important for invasion as it regulates the Tight Junction between the parasite and the host erythrocyte. While calcium is known to regulate parasite egress, in the present study, PfPK2 depletion did not affect parasite egress.

Collectively, present studies shed light on a novel signalling pathway in malaria parasite in which PfPK2 is an upstream regulator of key events like cGMP synthesis, which in turn is critical for calcium release. Given these second messengers are critical for host erythrocyte invasion, it is reasonable to conclude that PfPK2 regulates invasion by controlling the levels of these second messengers in the parasite, which in turn activate downstream events involved in key processes like microneme secretion (Fig 8). In addition, PfPK2 may also regulate the glideosome-actomyosin motor activity as its depletion perturbs phosphorylation of key proteins involved in this activity (Fig 6B and 6C, Table 1), although it needs further experimental validation.

## Discussion

Present studies demonstrate that PfPK2 is critical for host erythrocyte invasion by *P*. *falciparum*. PfPK2 seems to be present in compartments that are proximal to the micronemes and rhoptries but did not exhibit co-localization with proteins that reside in this organelle. The compartment in which it is present appears to be vesicular as indicated by its punctate staining and each merozoite seems to contain 2–3 PfPK2-positive puncta, which do not co-localize with apical organelles. It is possible that its localization to these vesicular structures is dynamic and it is trafficked to other locations transiently, which may allow it to target proteins at distinct locations. PfPK2 depletion resulted in a severe impairment in microneme secretion, which included the release of AMA-1. Its depletion also caused defects in secretion of other microneme protein EBA175, which suggested that PfPK2 may have a generic role in microneme release. The parasite utilizes multiple pathways for the invasion of host erythrocytes, it interacts with the sialic acid on glycophorin A via EBL family members like EBA-175 that are released from the micronemes [44,62]. Alternatively, it utilizes sialic acid independent pathways, which typically involve RH family members like RH5 [63]. Despite reduced EBA175 secretion, the attachment was almost unaltered in PfPK2-depleted parasites, which suggested that independent pathways like RH5-basiginin may operate effectively to facilitate its attachment.

In contrast, the entry of the parasite into the erythrocyte was significantly compromised and corroborated well with reduced AMA-1 secretion. Live cell imaging revealed that PfPK2-depleted parasites exhibited prolonged attachment to erythrocytes but failed to invade or enter the erythrocytes. However, characteristic deformation of the erythrocyte membrane and/or echinocytosis was observed. These defects share striking resemblance to observations made in studies in which AMA1 was depleted or parasites were treated with R1 peptide, which prevents interaction between AMA1 and RON2/4 [8,42,43]. IFA studies revealed that PfPK2 depleted merozoites that were attached to the erythrocyte surface exhibited either very low levels or absence of detectable AMA1 at the TJ whereas RON2 was unchanged (Fig 5C), which supported a possible role of PfPK2 in TJ formation.

It was interesting to note that a few PfPK2-loxP merozoites managed to invade the erythrocytes upon RAP-treatment. However, most of these rings either did not mature and/or turned pyknotic (Fig 2F and 2G). It is indeed possible that post invasion, sealing of erythrocyte membrane may be impaired due to issues like abrogated TJ formation etc. As a result, the environment for the newly formed rings inside the host erythrocyte was possibly not congenial for growth.

In order to understand the mechanism via which PfPK2 may regulate host cell invasion, comparative phosphoproteomics was performed to identify proteins which may be regulated by PfPK2 signalling. These studies resulted in identification of several putative targets which may be involved in processes that may be pertinent to PfPK2 function in invasion which included signalling, invasion and glideosome/actomyosin motor (Fig 6B and 6C, Table 1, S1 Dataset, 1.2). Previously, the role of PfGAP45 in host cell invasion has been demonstrated [50] and its phosphorylation may promote its function of actomyosin motor anchoring [51]. Several other proteins of the motor and glideosome complex like Myosin E, Myosin light chain, GAP40 and IMC proteins also exhibited altered phosphorylation in PfPK2-depleted parasites suggesting that it may directly or indirectly facilitate their phosphorylation. The glideosome complex facilitated motor activity is also be required for movement of the merozoite post-TJ formation from apical to the basal end [9]. It is possible that altered phosphorylation of PfGAP45 and other glideosome-actomyosin related targets may contribute to this process.

One of the features of comparative phosphoproteomic studies was the fact that several signalling proteins exhibited aberrant phosphorylation upon PfPK2 depletion: Adenylyl cyclase β and PfPKAc were hyper-phosphorylated, which is likely to be an indirect effect possibly caused by deregulation of a phosphatase. Incidentally PPM2 phosphorylation [27] was also modulated. Guanylyl cyclase (GCα)-which generates cGMP in the parasite especially in the blood stages- is involved in several important processes like egress and invasion via its downstream effector kinase PfPKG [27]. GCα phosphorylation at S1214 was attenuated upon PfPK2 depletion without change in its expression. cGMP formation was found to be impaired, which confirmed the involvement of PfPK2 in cGMP generation. As mentioned above, this residue is unique to GCα from *Plasmodium spp*. as it is in an insert of the N-domain (Fig 7A). Since this domain is important for ATP binding [27], phosphorylation of this insert may have a bearing on the function of ATPase domain. Interestingly, GCα was previously reported to be hyperphosphorylated at S674 upon depletion of protein phosphatase PfPP1 and cGMP levels were reduced upon depletion of this phosphatase [61]. These phosphorylation events also occur in the ATPase domain, which was reported to play a role in cGMP synthesis and function of PfGCα [27]. While the direct relevance of phosphorylation of these sites in GCα function remains to be established, these studies suggested that its phosphorylation may play a critical role in its function of cGMP generation. One of the major functions of cGMP-PfPKG signalling is to stimulate the release of calcium in the parasite, which in turn regulates these processes [26,27,65]. Indeed, PfPK2 depletion impaired cGMP levels in the parasite (Fig 7C). Furthermore, calcium release was also significantly reduced in PfPK2-depleted parasites, which corroborated well with reduced cGMP levels (Fig 7D). Given that calcium signalling is involved in invasion [21,65,67], it is reasonable to propose that one of the ways PfPK2 may regulate invasion may be by facilitating calcium release possibly by regulating cGMP signalling. While cGMP regulation of calcium release-which occurs via PfPKG- has been well established, the underlying mechanisms are less clear. In addition to its role in invasion, cGMP mediated calcium release regulates egress of merozoites [27]. However, in the present study we did not see significant defects in egress upon PfPK2 depletion. Previous studies in which PfGCα was conditionally knocked down revealed almost complete ablation of cGMP and calcium in the parasite that severely impaired egress [27]. Even though PfPK2-depletion significantly reduced cGMP and calcium release (Fig 7C and 7D) their residual amounts which remained in the parasite may be sufficient for the parasite egress and not for invasion. It is also possible that the impact of PfPK2 signalling via these molecules may be more on processes post-egress and more relevant for invasion.

PI(4,5)P2 is hydrolyzed by PLC to generate IP3 and DAG and IP3 regulates calcium release. GCβ and PKG have been shown to regulate PIP2 and PIP3 levels in *P*. *berghei* ookinetes, which was attributed to possible phosphorylation of PI4K and PI4P5K by PbPKG as their phosphorylation was compromised upon depletion of this kinase [26]. In present studies, PI4P-5K phosphorylation was also reduced in the phosphorylation in PfPK2-depleted parasites (Fig 6B, Table 1, S1 Dataset, 1.3). Therefore, it is possible that PfPK2 mediated cGMP generation has a bearing on PIP2 formation and/or metabolism, which contributes to calcium release in the parasite. It was interesting to note that some of the key targets of PfPK2 in the present study were also identified as putative targets of protein kinases PfPKG and PfPKA which have also been implicated in invasion [23,68]. However, in most cases the phosphorylation sites were different than the sites influenced by PfPK2. For instance, adenylyl cyclase β (ACβ) and several glideosome/IMC related proteins (coronin, IMC1g, GAP45) were also regulated by PfPKA phosphorylation [68]. In the case of PfPKG [23], ACβ, as well as GCα were altered in addition to coronin, IMC1g, GAP45, GAP40 and MyoE, which were also regulated by PfPK2. Therefore, it appears that these three kinases- PfPK2, PfPKG and PfPKA- may regulate invasion by regulating several common targets. Therefore, it is reasonable to suggest that their cumulative effect is responsible for efficient invasion by the parasite.

Present studies established the role of PfPK2 in host erythrocyte invasion and specifically in its ability to regulate late events in invasion possibly via TJ formation. A novel pathway was deciphered in which it is an upstream regulator of cGMP-calcium signalling axis, which is critical for invasion (Fig 8). PfPK2 can be an attractive drug target as its inhibition is likely to block a signalling cascade necessary for the parasite to establish infection in the human host.

## Materials and methods

### Expression of recombinant proteins

For the expression of recombinant proteins and subsequent kinase assays, *PfPK2* was amplified using primer 12/13 from a plasmid that contained custom synthesized codon optimized version of PfPK2. The amplicon was cloned in *BamHI* and *NotI* restriction sites of pET28a (+) vector. The deletion mutants of PfPK2 protein were generated either by site directed mutagenesis or overlapping PCR using primers indicated in S1 Table. Briefly, to generate K140M and ΔRD mutant of PfPK2 site directed mutagenesis was performed using primer 14/15 and 16/17, respectively. PfPK2 deletion mutant for ΔCD and ΔRD+ΔCD was generated using primer set 12/18 and 12/19, respectively. The final PCR product was cloned in pET28a vector under *BamHI* and *NotI* restriction site as mentioned above.

Recombinant proteins were expressed in BL-21 (DE3) RIL strain using 1 mM of IPTG at 18°C for 16 h. Subsequently, the bacterial culture was pelleted at 3500 rpm for 15 min at 4°C. Cells were resuspended in a solution containing 50mM potassium phosphate buffer pH 7.4, 200mM NaCl, 1% Triton X-100, 10% glycerol, 1mM β-mercaptoethanol, and protease inhibitors. Sonication was performed for 10 min on ice followed by centrifugation at 10,000 rpm for 45 min to separate the supernatant and pellet fraction. The supernatant was collected and used for recombinant protein purification either by using AKTA FPLC or on His-Trap columns (GE Healthcare) as per the manufacturer guidelines or by manually purifying pre-equilibrated Ni-NTA-agarose (Qiagen). Purified proteins were eluted with imadzole and dialyzed overnight at 4°C against 50 mM Tris, pH 7.4, 10% glycerol, and 1 mM DTT and electrophoresed on a SDS-PAGE gel.

### Kinase assays

Tyically, protein kinase assays were performed using recombinant ~0.1 μM 6xHis-PfPK2 in a buffer containing 50 mM Tris pH 7.5, 10 mM magnesium chloride, 1 mM DTT and 100 μM [γ-^32^P] ATP (6000 Ci/mmol) (Perkin Elmer). In some experiments, 0.1–5 μM calmodulin in the presence of 10 μM CaCl_2_ or 100 μM EGTA was used and ~2 μg of Histone IIa was used as phosphoacceptor substrate. Typically, the reactions were carried out at 30°C for 45 min in a water bath and were stopped by boiling the reaction mix in SDS-PAGE sample buffer. The samples were subjected to SDS-PAGE, the gel was subsequently dried and exposed to a phosphorimager image plate. The phosphorylation of the substrate was then detected by phosphorimaging using an Amersham Typhoon scanner (GE Healthcare). Equal amount of PfPK2 and various mutants were used to compare their activity in assays which were performed as described above.

#### Parasite cultures

*Plasmodium falciparum* strains 1G5DC was obtained from European Malaria Reagent Repository (EMRR) and DSM1 was obtained from *BEI* resources, Malaria Research and Reference Reagents Resource Centre (MR4), American Type Culture Collection (ATCC). Parasites were maintained in O+ human erythrocytes (5% hematocrit) in RPMI-1640 supplemented with 0.5% Albumax II and 50 μg/mL hypoxanthine [69]. Cultures were maintained at 37°C under a mixture of 5% CO_2_, 3% O_2_, and 91.8% N_2_ or 5% CO_2_. Fresh erythrocytes were used to dilute parasite with fresh culture media to maintain 3–5% parasitemia at 5% haematocrit. For culturing various transgenic lines relevant drugs were used as described below. Parasite synchronization was carried out using sorbitol as described previously [70].

#### Percoll purification

*P*. *falciparum* infected erythrocytes (containing ~5% schizonts) were pelleted down and resuspended in 2 ml complete medium. 3 ml of 70% percoll was used and infected-erythrocytes were gently poured alongside the rim of the tube. The samples were centrifuged in a swinging bucket rotor for 10 min at 2000 rpm (RT) without brake. The parasites which were mainly enriched schizonts, were gently removed and washed once with culture medium and added to a new flask containing fresh erythrocytes at 2% hematocrit.

### Plasmid construction and transfection into 1G5DC parasites

#### 1G5DC+pSLI-PfPK2-loxP

All PCR primers used in this study were synthesized by Sigma and are indicated Supplementary Table S1.

A transgenic parasite line using dimerazable Cre recombinase (diCre) was generated to conditionally knockout PfPK2. These parasites were generated in 1G5DC background as these parasites express diCre. A selection linked integration (SLI) approach was employed for this purpose [36]. For this purpose, a 330bp homology arm corresponding to 5’-end of *PfPK2* gene was amplified using primers 1/2 and cloned in *NotI* and *PmeI* sites upstream of loxP and Myc-tag coding sequence in pSLI-N-sandwich-loxP(K13) vector, which was a gift from Tobias Spielmann (Addgene plasmid #85793; http://n2t.net/addgene:85793; RRID:Addgene_85793). A recodonized version of full-length PfPK2 with at *AvrII* and *StuI* sites upstream of a loxP sequence in frame with GFP tag was custom synthesized as a G-block (Genscript). The resulting plasmid DNA construct (pSLI-PfPK2-loxP) was transfected in 1G5DC *P*. *falciparum* strain [35]. Transfections were performed using ~100μg of purified plasmid DNA constructs in uninfected erythrocytes followed by the addition of percoll-enriched schizont stage parasites. Subsequently, transgenic parasites were selected by using 2nM WR99210 and recombinants were enriched using 1.5μM DSM-1.

Drug selected parasites were subjected to genotyping followed to assess the modification of the desired locus. After limited dilution cloning of drug resistant parasites, a clone was obtained, which lacked the endogenous unmodified locus was used for further analysis. PCR for genotyping of was performed followed by Sanger sequencing for the PCR products to confirm the desired modifications. Rapamycin was added to dimerize the Di-Cre recombinase in 1G5DC/PfPK2-loxP transgenic line resulting in the excision of loxP flanked region in the modified PfPK2 genomic locus.

#### HA-cPfPK2-PK2-loxP

In the above-mentioned PfPK2-loxP parasites, PfPK2 was overexpressed to complement the effects of PfPK2 depletion. For this purpose, PfPK2 cDNA was prepared from *P*. *falciparum* 3D7 RNA (isolated using QIAGEN RNeasy kit) and PCR amplification was done using primers 10/11 to amplify PfPK2 with an N-terminal HA tag and primers 8/9 was used to mutate the internal *KpnI* site in PfPK2 by overlapping PCR. The PCR product was cloned in pARL-BSD vector (a kind gift from Dr. Asif Mohmmed) using *KpnI* and *AvrII* restriction sites. The plasmid DNA was transfected as described below, followed by selection using 2.5μg/ml of blasticidin and 1.5 μM DSM1.

### Growth rate and invasion assays

PfPK2-loxP parasites were cultured in the presence of 5 nM WR99210 and 1.5 μM DSM1. Subsequently, for growth rate assay, ring stage synchronized parasites were seeded at ~0.5–1% parasitemia at 2% hematocrit. 200 nM rapamycin was added to parasite cultures for ~6–8 hours to deplete PfPK2. Parasite growth was monitored periodically by microscopic analysis of Giemsa-stained thin blood smears and counting individual parasitic stages.

For flow cytometry based assays, samples were collected at desired time and fixed with 1% PFA/0.0075% glutaraldehyde and kept on an end-to-end rocker for 15 min. After completion, samples were either stored at 4°C or processed directly for Hoechst 33342 staining for 10 min at 37°C. Samples were then washed at least twice with FACS buffer (0.3% BSA + 0.02% Sodium Azide) followed by analysis [71,72] on BD verse (BD biosciences).

The parasite invasion assays were carried out as described previously with slight modifications [21,71,73]. Schizonts were washed twice and resuspended in culture medium with fresh erythrocytes to achieve 2% hematocrit. Invasion experiments were performed using 0.5–1% schizont parasitemia and 2% hematocrit in a 6-well plate in a gas chamber equilibrated with gas mixture described above at 37°C and samples were collected periodically for upto ~12 hours. Thin blood smears were Giemsa-stained and the number of schizonts or newly formed rings was counted. Alternatively, flow cytometry was used as described previously [71,73] for which parasites were fixed using 1% PFA + 0.0075% glutaraldehyde in either ALS solution or in FACS buffer followed by staining the parasite nuclei using Hoechst 33342. Flow cytometry data was acquired using BD FACS verse and analysed using FlowJo V.10 software (Tree Star, Ashland, OR).

### Live-cell imaging

Time-lapse imaging was performed as described previously with slight modifications [73]. Briefly, PfPK2-loxP parasites were synchronized at the ring stage and treated with DMSO or rapamycin for 6h and were allowed to develop to schizont stage. Schizonts purified and were cultured in the presence of 25nM PfPKG-inhibitor ML10 [74,75] with fresh erythrocytes at 2% hematocrit in RPMI1640 complete media. Subsequently, ML10 was washed after 4-6h and parasites were plated on glass bottom chamber slides (Ibidi) coated with 0.5 mg/ml concanavalin A. The slides were kept at 37°C, supplied with a humidified environment under 5% CO_2_ and imaging was performed using a Zeiss Axio Observer microscope. Images were typically taken every 2s for at least 10 min, and resulting time-lapse videos were processed using Axio Vision 4.8.2 analysis software.

### Parasite attachment assay

The parasite-erythrocyte attachment assays were carried out as previously described with minor changes [21,30]. PfPK2-loxP parasites were synchronized at ring stage (~3% parasitemia, 2% hematocrit) and treated with DMSO and Rapamycin as described above and were allowed to mature to schizont stage (~44 hpi). Subsequently, 25nM ML10, which prevents egress was added for 6h, to allow maturation of schizonts and merozoites were released subsequent to the washing of the inhibitor [75]. Assays were performed in 6-well culture plates containing fresh erythrocytes treated either with 1 mM cytochalasin D or DMSO (control) with shaking at 80 rpm at 37°C. After 8–12 h, thin blood smears were stained with Giemsa. The number of parasites attached to erythrocytes was quantified by microscopically examining the stained parasites. In some experiments, IFA was performed to visualize the tight junction as described below.

### Phosphoproteomics and mass spectrometry

#### Proteomics studies

*Cell lysis and protein extraction and TMT labeling*. The 1G5DC or PfPK2-loxP parasites were cultured and synchronized using sorbitol. Ring stage parasites were treated with either DMSO or RAP for duration of 6 hours and experiments were done using three independent biological replicates. The parasites were subsequently harvested upon maturation to schizonts, which was done ~44 hours post-infection, by lysing the infected red blood cells with saponin. The resulting parasite pellet was washed and dissolved in protein extraction buffer (50 mM triethylammonium bicarbonate (TEABC), 2M SDS, and 1X protease and phosphatase inhibitors). The samples were sonicated and centrifuged, and the resulting supernatant was used for protein estimation by bicinchoninic acid assay. Equal amounts of protein were taken from both DMSO and RAP-treated parasites and subjected to reduction with 20 mM dithiothreitol (DTT) at 60°C for 30 minutes, followed by alkylation with 20 mM iodoacetamide for 10 minutes in dark at ambient temperature. Subsequently, proteins were digested using a trypsin and lys-C mix (Promega, Madison, USA) at a ratio of 100:1, and tryptic peptides were cleaned using a C18 column and dried with a SpeedVac concentrator (Thermo Fisher Scientific, USA). The peptides were quantified using the Pierce Quantitative Colorimetric Peptide Assay Kit. Subsequently, 500 μg of peptides from each sample were taken and processed for TMT labelling as per manufacturer’s instructions. After confirming a labelling level of >95%, the reaction was quenched by adding 8 μl of hydroxylamine. The labelled peptides were pooled, dried, and cleaned using C18 Sep-Pak (Waters, Milford, MA, USA).

*Phosphopeptide enrichment and fractionation*. A 50μg aliquot of the sample was used for total proteome analysis, and the remaining sample was subjected to phosphopeptide enrichment. For the first phosphopeptide enrichment step, we used the TiO2 Phosphopeptide Enrichment Kit (Thermo Scientific, Catalog number: 88303), following manufacturer’s instructions. The flow-through collected during this step was used for the second phosphopeptide enrichment step, utilizing the High-Select Fe-NTA Phosphopeptide Enrichment (Catalog number: A32992). The eluent from both phosphopeptide enrichment steps was combined for fractionation. To resolve complexity, we employed C18 stage tip fractionation, collecting a total of 24 fractions that were subsequently concatenated into six fractions. The peptide samples were dried and dissolved in 0.1% formic acid prior to mass spectrometry analysis.

*Mass spectrometric analysis*. Mass spectrometry analysis was performed using an Orbitrap Fusion Tribrid mass spectrometer coupled to an Easy-nLC 1200 nano-flow UPLC system (Thermo Fischer Scientific, Germany). All fractions were loaded onto a trap column nanoViper (2 cm, 3 μm C18 Aq) (Thermo Fischer Scientific). Subsequently, eluted peptides were further resolved on an analytical column (75 μm × 15 cm, C18, 2 μm particle size) (Thermo Fischer Scientific). A gradient of 5–35% solvent B (80% acetonitrile in 0.1% formic acid) was used over a 120-minute, at a flow rate of 300 nl/min.

Data were acquired in data-dependent acquisition (DDA) mode. Precursor ions were acquired in full MS scan mode in the range of 400–1600 m/z, with an Orbitrap mass analyzer resolution of 120,000 at 200 m/z. An automatic gain control (AGC) target value of 2 million with an injection time of 50 ms and dynamic exclusion of 30 seconds was used. The selection of the most intense precursor ions was performed at top speed data-dependent mode, isolated using a quadrupole with an isolation window of 2 m/z and an isolation offset of 0.5 m/z. Subsequently, filtered precursor ions were fragmented using higher-energy collision-induced dissociation (HCD) with 34±3% normalized collision energy. MS/MS scans were acquired in the range of 110–2000 m/z in the Orbitrap mass analyzer at a mass resolution of 60,000 mass resolution/200 m/z. AGC target was set to 100,000 with an injection time of 54 ms. For each condition, MS/MS data were acquired in duplicates.

#### Data analysis

MS/MS raw data was used to search against a combined protein database of *P*. *falciparum* 3D7 (downloaded from PlasmoDB web resource, version 46) and humans using SEQUEST and Mascot (Version 2.4.1) via Proteome Discoverer v2.2 (Thermo Fisher Scientific). Trypsin was selected as the protease of choice with a maximum of two missed cleavages. A precursor mass tolerance and fragment mass tolerance of 10 ppm and 0.02 Da were selected, respectively and the data was filtered at 1% FDR at PSM level. Static modifications were carbamidomethylation at cysteine and TMT labeling at lysine and N-terminus of peptide. For dynamic modifications, methionine oxidation, acetylation at N-terminus of protein and S/T/Y phosphorylation were selected. The phosphorylation fold changes were normalized against the total proteome data as mentioned earlier [76]. The ptmRS node was used during the search to determine the probability of phosphorylation site localization and a ptmRS score ≥99% was used as a cut off.

Phosphopeptides along with their fold changes (RAP/DMSO) from three biological replicates were uploaded on Perseus software (Version 1.4) to determine the p-value using student’s t-test for each phosphosite and p<0.05 was considered as significant (S1 Dataset for PfPK2-loxP parasites and S2 Dataset for 1G5DC control line). In addition, fold change cut-off of 1.2 was applied to further filter differentially phosphorylated sites [21]. Phosphosites that were identified differentially phosphorylated in two or more biological replicates were considered for the further analysis. The prediction of protein-protein interactions was performed using, STRING web resource (Version 11.5) was used. Proteins that were found to be differentially phosphorylated were considered for the analysis, and interactions were predicted with medium confidence filter (Szklarczyk et al, 2011).

#### Immunofluorescence Assay (IFAs)

Immunofluorescence assays (IFA) were performed either on parasite suspensions or thin blood smears as previously described [77]. For IFA on cell suspension, the parasite pellet was washed in 1x PBS to remove the culture medium and fixed for 30 min using 4% PFA and 0.0075% glutaraldehyde prepared in PBS. For permeabilization, 0.1% Triton-X100 was used for 15 min. For air-dried thin blood smears, cold methanol and acetone mix (1:1, v:v) was used for 2 min followed by blocking with 3% BSA for 45 min at room temperature was used for blocking followed by incubation with primary antibody for 12h at 4°C. After washing, Alexafluor mouse/rabbit 488/594-labeled secondary antibodies were added (Invitrogen) for 2h at ambient temperature. Samples were mounted using Vectashield mounting media (Vector Laboratories Inc.) which contained DAPI to label nuclei. The stained parasites were visualized using either Axio Imager Z1 microscope or LSM980 confocal microscope (Carl Zeiss). The processing of images was done using AxioVision 4.8.2 or Zeiss ZEN black/blue software. Z-stacks that best represented the immunolocalization were used for illustrations in the figures.

#### Immunoblotting

Parasites were collected and infected erythrocytes were lysed using 0.05% saponin (w/v) on ice for 10 min. Centrifugation at 8000 rpm was done to isolate the parasite pellet, which was washed three times with pre-chilled PBS. The parasite pellet was re-suspended in lysis buffer (10 mM Tris pH 7.5, 100 mM NaCl, 5 mM EDTA, 1% Triton X-100, and complete protease inhibitor cocktail (Roche Applied Science) or 2% SDS and homogenized by either passing the solution through a 26-gauge needle or by sonication. The supernatant from the centrifugation of the lysates at 14,000 g for 30 min at 4°C was used to estimate the amount of protein using a BCA protein estimation kit (Pierce). After separation by SDS-PAGE, lysate proteins were transferred to a nitrocellulose membrane. Immunoblotting was performed as described previously [21,73] using specific antisera and blots were developed using SuperSignal West Pico or Dura Chemiluminescence Substrate (Thermo Scientific) following manufacturer’s instructions.

#### Detection of parasite secreted proteins

PfPK2-loxP parasites were synchronized tightly at ring stage and treated with DMSO and rapamycin followed and were allowed to mature to schizonts (42–44 hpi) and secrete microneme and rhoptry proteins were detected in the extracellular milieu as described previously [21,73]. The residual supernatant was used for Western blot analysis with antibodies against several proteins of interest, as mentioned below.

#### Determination of calcium release in the parasite

Calcium release in the parasite was measured as described previously with brief modifications [27]. ~1x10^7^ percoll purified schizonts from DMSO- or RAP- treated cultures were incubated in a phenol red-free RPMI containing 10μM Fluo4-AM (Invitrogen, Carlsbad, CA) in dark at 37°C for 45 min. Parasites were washed with phenol red-free RPMI and after 20 min 100μl of parasite suspension was transferred to a 96-well plate. As a control, in some wells only phenol red-free RPMI was included. The fluorescence was measured at 22-sec intervals for 5 min using a Synergy H1 microplate reader (BioTek) with excitation and emission wavelengths of 483 and 525 nm, respectively. Parasites were resuspended and transferred to wells containing 1.5% DMSO or 75μM zaprinast or 20μM A23187 (20μM) and fluorescence was measured as described above. The average of the relative fluorescence units at each time point and condition was determined after subtracting baseline and DMSO control values.

#### Measurement of intracellular cyclic GMP levels

Intracellular cyclic nucleotide levels in mature segmented schizonts were measured using enzyme-linked immunosorbent assay (ELISA)-based high-sensitivity cGMP assay kit as suggested by the manufacturer (Cell Signalling Technology, Cat No. #4360). Briefly, ~10^7^ percoll-purified schizonts were obtained from RAP- or DMSO-treated cultures. The purified schizonts were incubated for 30 min in culture medium containing ML10 in the presence of zaprinast (100 μM) [27]. Parasites were pelleted at 9,000 × g, washed with 1x PBS followed by centrifugation at 9,000 × g, and the pellet was collected and stored at –80°C until required. Parasites were lysed using cell lysis buffer, as per the manufacturer’s instruction and standards were also prepared in the same buffer. 50μl samples and 50μl HRP-linked cGMP solution was added to the cGMP assay plate and incubated for 3h on horizontal orbital plate shaker at room temperature followed by 4 washes with 200 μl wash buffer. Further incubations were performed in 100 μl of TMB substrate for 30 min at ambient temperature. Reaction was terminated by adding 100 μl STOP solution and absorbance was measured at 450 nm using a Synergy H1 microplate reader (BioTek).

#### Densitometry and statistical analysis

Image J (NIH) software was used to perform densitometry of Western blots. The band intensity of the loading control was used for normalization. Statistical analysis was performed using Prism (Graph Pad software Inc USA). Data was generally represented as mean ± Standard error of mean (SEM), unless indicated otherwise and p < 0.05 was considered as statistically significant. Unless indicated otherwise, most experiments were done at least three times.

## Supporting information

S1 Fig Expression and purification of recombinant PfPK2 and kinase assay. **A.** Coomassie-stained SDS-PAGE gel showing recombinant 6xHis tagged PfPK2 or its mutants lacking the RD and or the CD domains which were expressed and purified as indicated in Methods section. **B.** Coommassie stained gel of the kinase assay described in Fig 1D. The corresponding phosphorimage is provided in Fig 1D.
(PDF)

S2 Fig RAP addition does not affect 1G5DC parasites. Growth rate assays were performed using control 1G5DC parasites along with PfPK2-loxP parasites as described in Fig 2E in the presence of DMSO or RAP. After 6h, RAP was washed and parasite growth was assessed after each cycle by performing flow cytometry (SEM ± SE, n = 3, ANOVA, ns—not significant).
(PDF)

S3 Fig Localization of PfPK2. **A.** IFA was performed on thin blood smears of PfPK2-loxP parasites using anti-GFP to detect PfPK2 and co-stained with antibodies against proteins that localize to various subcellular compartments: MSP1 (parasite plasma membrane), AMA1 (micronemes), and RhopH3 (Rhoptries). **B.** IFA was performed on PfPK2-loxP parasites using anti-GFP and anti-GAP45 antibodies upon DMSO or RAP treatment. IFA revealed that PfPK2 is present in punctate structures in parasite and was depleted upon RAP treatment.
(PDF)

S4 Fig Effect of PfPK2 depletion on parasite division and reversal of phenotypic defects upon episomal expression of PfPK2. **A.** Western blot was performed on lysates prepared from cHA-PfPK2-loxP parasites, which episomally express HA-PfPK2 after DMSO or RAP treatment using anti-GFP, anti-HA or anti-BiP (loading control) antibodies. There was no loss of HA-PfPK2 whereas GFP-PfPK2 was depleted upon RAP-treatment. **B.** PfPK2-loxP parasites were treated with either DMSO or RAP at ring stages. After ~40–44 h.p.i parasites were treated with E64, and mature segmentor stage parasites were traced. Giemsa-stained blood smears were prepared and number of merozoites per schizont were counted and % schizonts possessing indicated number of merozoites was determined (SEM ± SE, n = 2, ANOVA, P>0.05; ns -not significant). **C.** PfPK2-loxP or cHA-PfPK2-loxP parasites were treated with DMSO or RAP and the number of abnormal/pyknotic parasites upon RAP addition were counted after 72 hpi (as described for Fig 2G). A significant number of abnormal parasites were observed in PfPK2-loxP upon RAP addition whereas the change observed in cHA-PfPK2-loxP parasites was not significant (SEM ± SE, n = 3, ANOVA, ** P<0.01, ns -not significant). **D.** Invasion assays were performed as described in Fig 3A, for indicated lines in the presence or absence of treatment of schizonts with DMSO or RAP and the number of rings were counted after 12h, which reflected invasion. Fold change in invasion upon RAP treatment was determined (SEM ± SE, n = 3, ANOVA, *** P<0.001, ns P>0.05; ns -not significant.
(PDF)

S5 Fig PfPK2 is not expressed in gametocytes. PfPK2-loxP gametocytes of different stages were stained with anti-Pfs16 and anti-GFP (to detect PfPK2) antibodies. No detectable expression in sexual stages was observed upon PfPK2 depletion.
(PDF)

S6 Fig Secretion of microneme and rhoptry proteins upon PfPK2 depletion. DMSO or RAP treated PfPK2-loxP schizonts were cultured for one cycle and the release of AMA1 (A), EBA175 (B) and RhopH3 (C) from parasites was determined by performing Western blotting on culture supernatant using specific antibodies. Western blots were also performed on total parasite protein lysates, which were used for normalization of secreted proteins. The secretion of these proteins in the supernatant was quantitated by densitometry of the Western blot provided in Fig 5A.
(PDF)

S7 Fig IFA was performed on PfPK2-loxP parasites using anti-EBA175 and anti-AMA1 antibodies upon DMSO or RAP treatment. (PDF)

S8 Fig Sequence comparison between the ATPase domain of Human (ATP8A1 and ATP8A2) with this domain present in GCα of *Toxoplasma gondii*, *Plasmodium berghei*, *Plasmodium falciparum*, *Plasmodium vivax* suggested that S1214 which was hypophosphorylated upon PfPK2 depletion (Fig 7A and 7B) is only conserved in *Plasmodium* spp., long inserts are present in the case of *Plasmodium spp*. and S1214 is present in one of these inserts. (PDF)

S1 Table PCR Primers used in this study. (PDF)

S1 Video Time-lapse video microscopy of DMSO-treated PfPK2-loxP parasites (related to Fig 4Aa). (AVI)

S2 Video Time-lapse video microscopy of RAP- treated PfPK2-loxP parasites (related to Fig 4Ab). (AVI)

S3 Video Time-lapse video microscopy of RAP- treated PfPK2-loxP parasites (related to Fig 4Ac). (AVI)

S4 Video Time-lapse video microscopy of RAP- treated PfPK2-loxP parasites (related to Fig 4Ad). (AVI)

S1 Dataset Phosphoproteomics dataset on PfPK2-loxP parasites. **1.1:** List of phopshopeptides identified in the phosphoproteomic analysis of PfPK2 mutant parasite. **1.2:** List of hyperphopshopeptides/sites identified in the phosphoproteomic analysis of PfPK2 parasite. **1.3:** List of hypophopshopeptides/sites identified in the phosphoproteomic analysis of PfPK2 parasite. **1.4:** Details of TMT labels used for phosphoproteomic analysis of PfPK2 mutant parasite. **1.5:** Protein-protein interaction analysis of differentially phosphorylated proteins identified in PfPK2 mutant parasite.
(XLSX)

S2 Dataset Phosphoproteomics dataset on control 1G5DC parasites. **2.1:** List of phopshopeptides identified in the phosphoproteomic analysis of 1G5DC control parasite. **2.2:** List of hyperphopshopeptides/sites identified in the phosphoproteomic analysis of 1G5DC control parasite. **2.3:** List of hypophopshopeptides/sites identified in the phosphoproteomic analysis of 1G5DC control parasite. **2.4:** Details of TMT labels used for quantitative phosphoproteomic analysis of 1G5DC control parasite.
(XLSX)
