# Supplementary figures and images for: Protein kinase PfPK2 mediated signalling is critical for host erythrocyte invasion by malaria parasite
Source: PLoS Pathog. 2023 Nov 21;19(11):e1011770. doi: 10.1371/journal.ppat.1011770 (PMC10662742; doi:10.1371/journal.ppat.1011770)

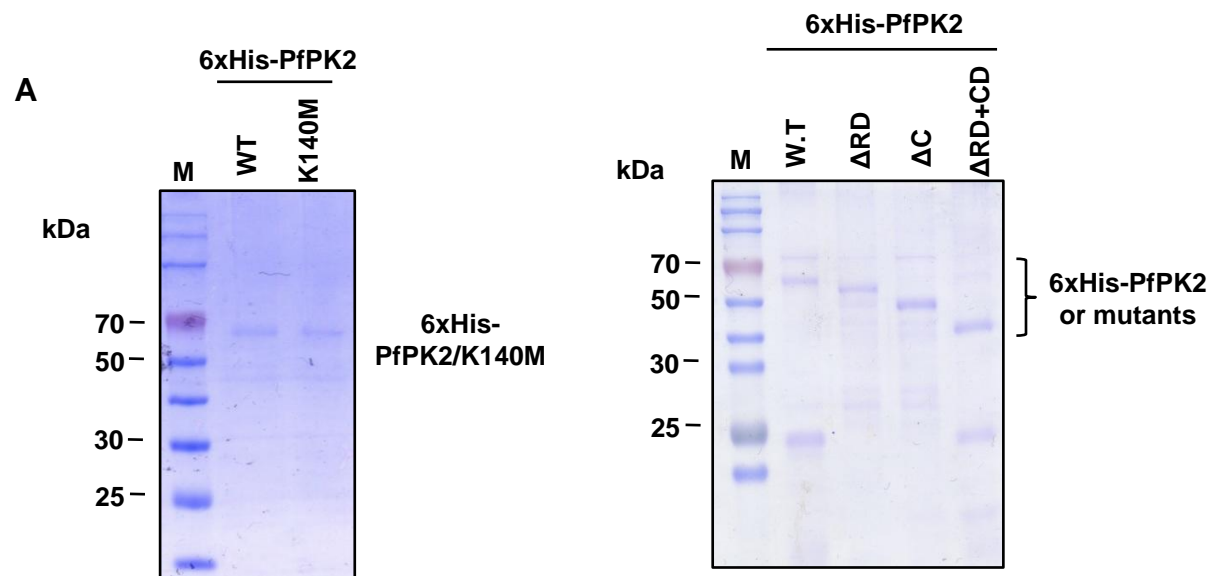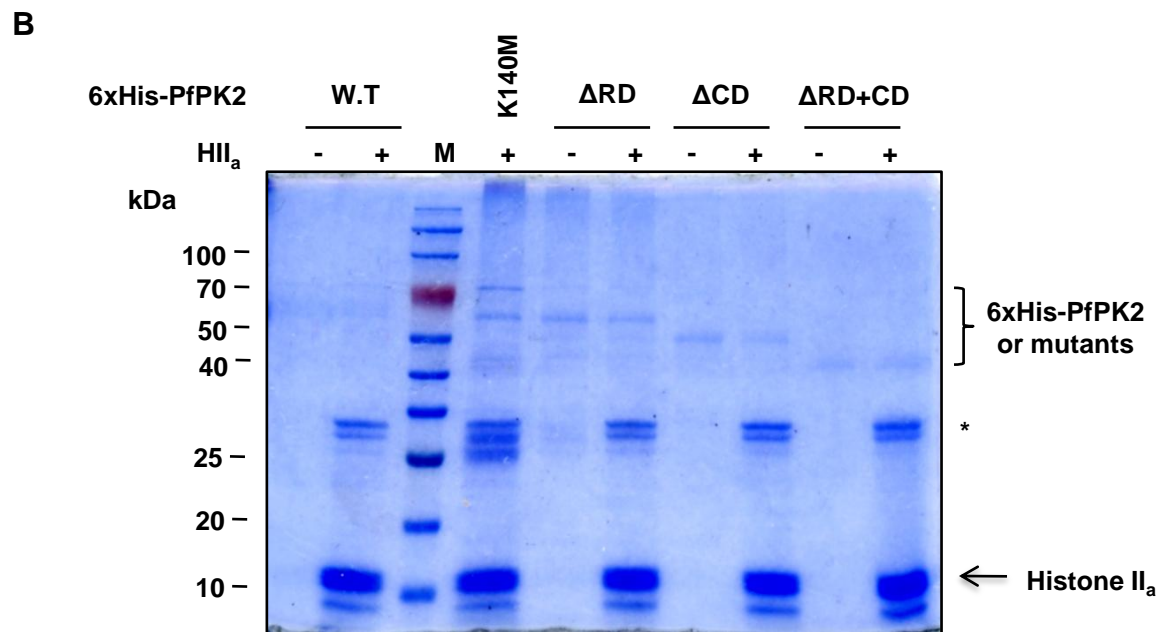

Supplement: S1 Fig — A. Coomassie-stained SDS-PAGE gel showing recombinant 6xHis tagged PfPK2 or its mutants lacking the RD and or the CD domains which were expressed and purified as indicated in Methods section. B. Coommassie stained gel of the kinase assay described in Fig 1D. The corresponding phosphorimage is provided in Fig 1D. (PDF) [file ppat.1011770.s001.pdf]

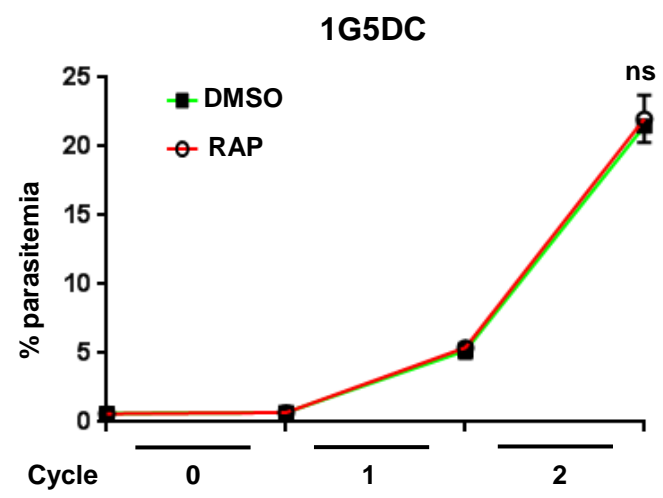

Supplement: S2 Fig — Growth rate assays were performed using control 1G5DC parasites along with PfPK2-loxP parasites as described in Fig 2E in the presence of DMSO or RAP. After 6h, RAP was washed and parasite growth was assessed after each cycle by performing flow cytometry (SEM ± SE, n = 3, ANOVA, ns—not significant). (PDF) [file ppat.1011770.s002.pdf]

A

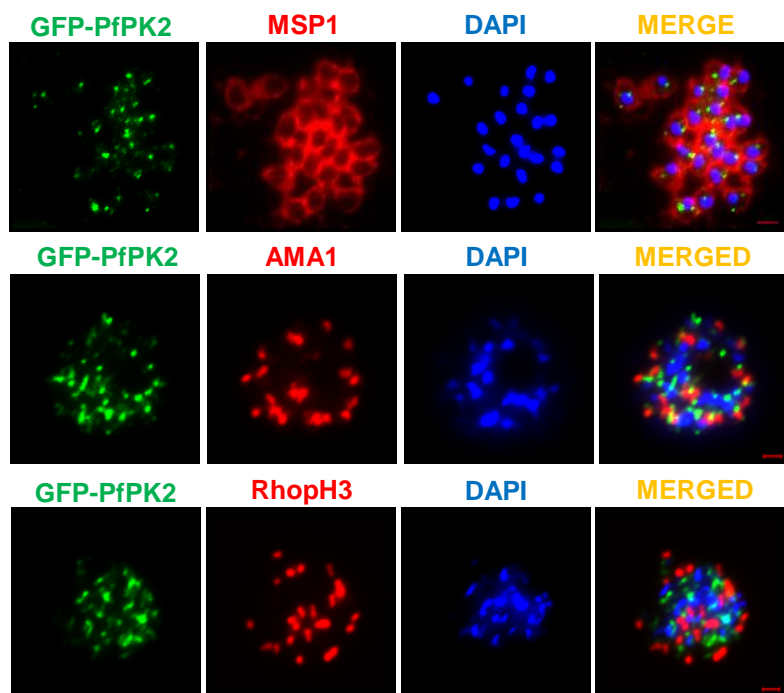

B

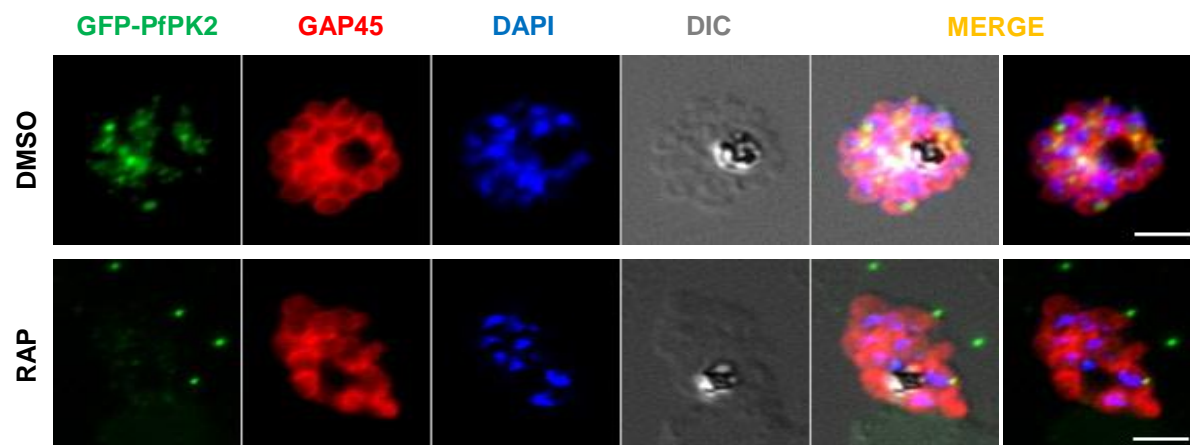

Supplement: S3 Fig — A. IFA was performed on thin blood smears of PfPK2-loxP parasites using anti-GFP to detect PfPK2 and co-stained with antibodies against proteins that localize to various subcellular compartments: MSP1 (parasite plasma membrane), AMA1 (micronemes), and RhopH3 (Rhoptries). B. IFA was performed on PfPK2-loxP parasites using anti-GFP and anti-GAP45 antibodies upon DMSO or RAP treatment. IFA revealed that PfPK2 is present in punctate structures in parasite and was depleted upon RAP treatment. (PDF) [file ppat.1011770.s003.pdf]

A

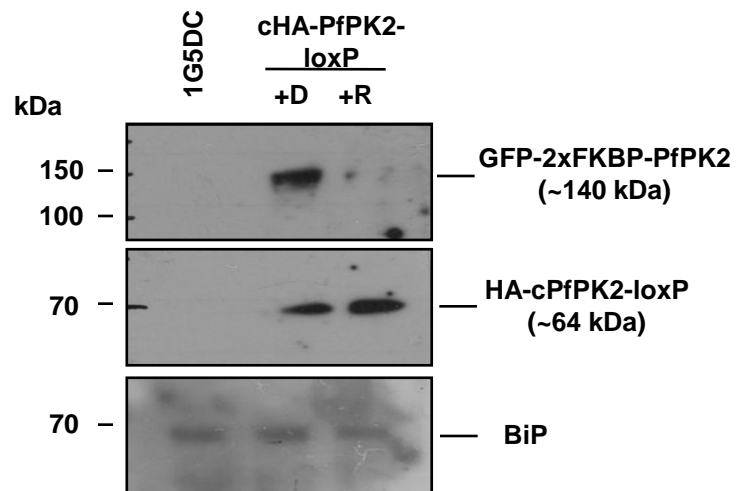

B

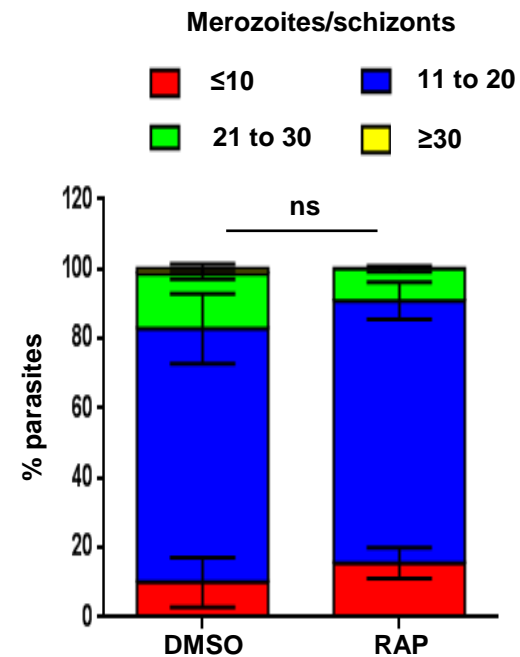

C

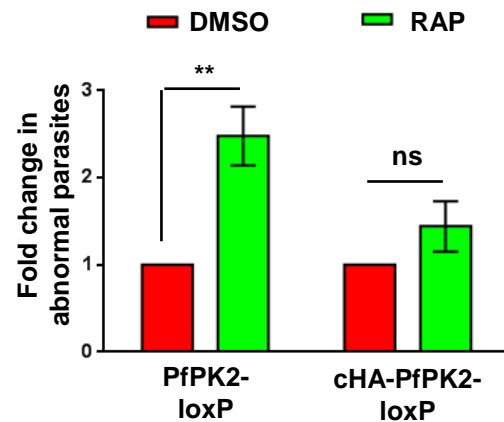

D

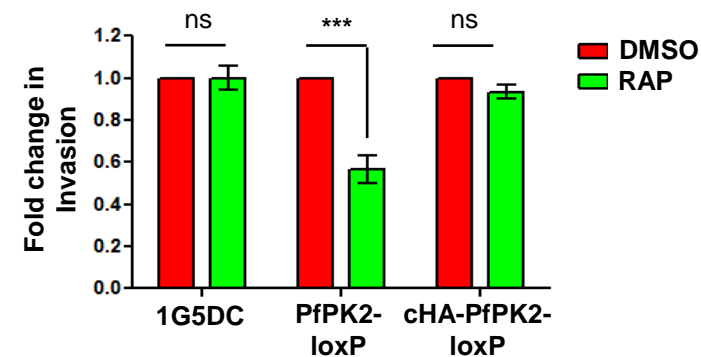

Supplement: S4 Fig — A. Western blot was performed on lysates prepared from cHA-PfPK2-loxP parasites, which episomally express HA-PfPK2 after DMSO or RAP treatment using anti-GFP, anti-HA or anti-BiP (loading control) antibodies. There was no loss of HA-PfPK2 whereas GFP-PfPK2 was depleted upon RAP-treatment. B. PfPK2-loxP parasites were treated with either DMSO or RAP at ring stages. After ~40–44 h.p.i parasites were treated with E64, and mature segmentor stage parasites were traced. Giemsa-stained blood smears were prepared and number of merozoites per schizont were counted and % schizonts possessing indicated number of merozoites was determined (SEM ± SE, n = 2, ANOVA, P>0.05; ns -not significant). C. PfPK2-loxP or cHA-PfPK2-loxP parasites were treated with DMSO or RAP and the number of abnormal/pyknotic parasites upon RAP addition were counted after 72 hpi (as described for Fig 2G). A significant number of abnormal parasites were observed in PfPK2-loxP upon RAP addition whereas the change observed in cHA-PfPK2-loxP parasites was not significant (SEM ± SE, n = 3, ANOVA, ** P<0.01, ns -not significant). D. Invasion assays were performed as described in Fig 3A, for indicated lines in the presence or absence of treatment of schizonts with DMSO or RAP and the number of rings were counted after 12h, which reflected invasion. Fold change in invasion upon RAP treatment was determined (SEM ± SE, n = 3, ANOVA, *** P<0.001, ns P>0.05; ns -not significant. (PDF) [file ppat.1011770.s004.pdf]

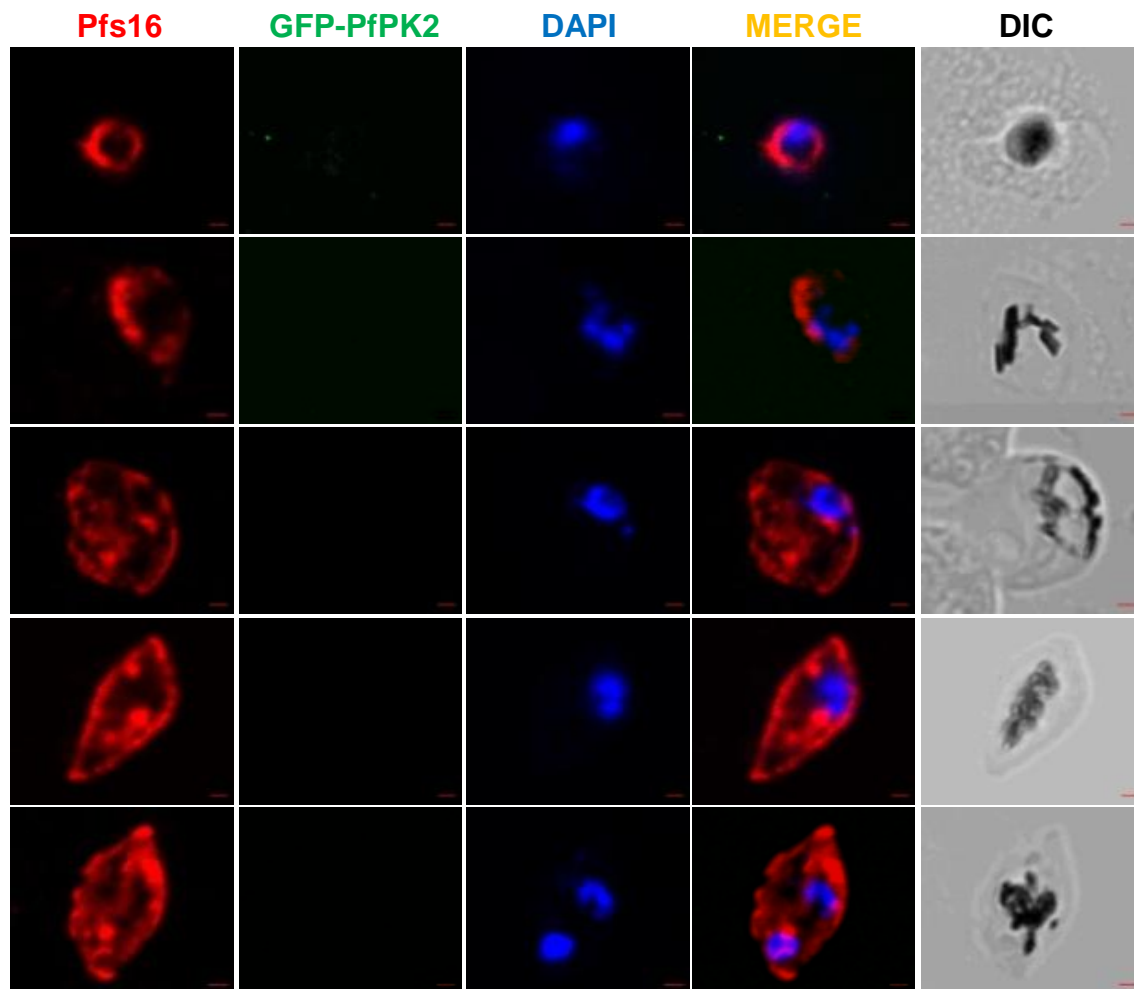

Supplement: S5 Fig — PfPK2-loxP gametocytes of different stages were stained with anti-Pfs16 and anti-GFP (to detect PfPK2) antibodies. No detectable expression in sexual stages was observed upon PfPK2 depletion. (PDF) [file ppat.1011770.s005.pdf]

**A**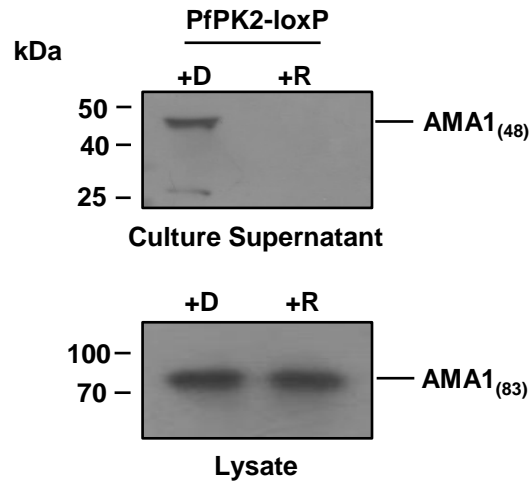**C**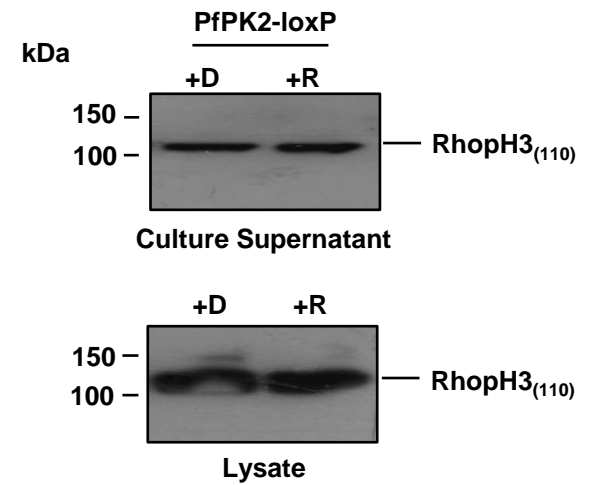**B**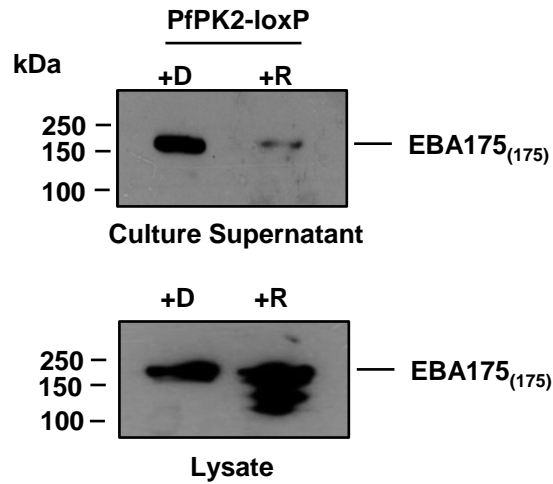

Supplement: S6 Fig — DMSO or RAP treated PfPK2-loxP schizonts were cultured for one cycle and the release of AMA1 (A), EBA175 (B) and RhopH3 (C) from parasites was determined by performing Western blotting on culture supernatant using specific antibodies. Western blots were also performed on total parasite protein lysates, which were used for normalization of secreted proteins. The secretion of these proteins in the supernatant was quantitated by densitometry of the Western blot provided in Fig 5A. (PDF) [file ppat.1011770.s006.pdf]

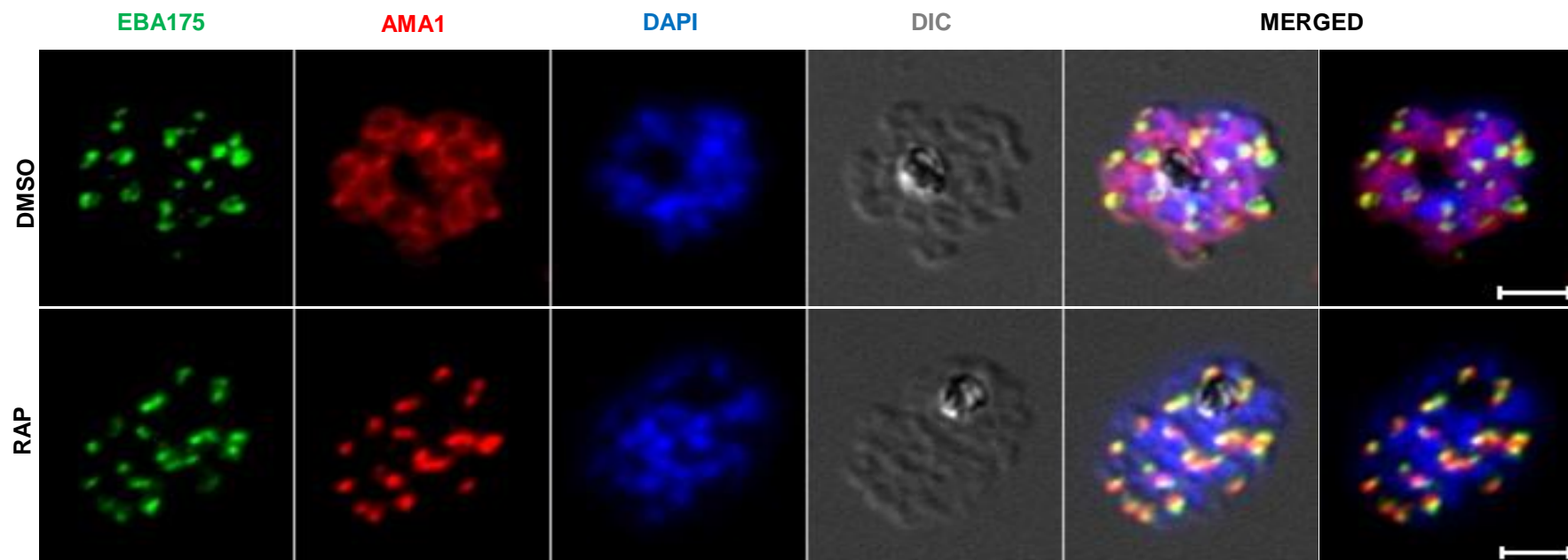

Supplement: S7 Fig — (PDF) [file ppat.1011770.s007.pdf]
