# Supplementary material for: Protein kinase PfPK2 mediated signalling is critical for host erythrocyte invasion by malaria parasite
Source: PLoS Pathog. 2023 Nov 21;19(11):e1011770. doi: 10.1371/journal.ppat.1011770 (PMC10662742; doi:10.1371/journal.ppat.1011770)
Supplement: S8 Fig — (PDF) [file ppat.1011770.s008.pdf]

```
PbGCα        KNLAHNSSRHSRIGSNHFERYNNELKKNTYTDINKKNNEINSYVSHENT-----------     1131
PfGCα        KHLTYTNSRSSKGISNNMDRYNINPTTTTTNNN-NNNNNINNINNINNNNNNNNNNNNNK     1258
PvGCα        KNLTHFPSRSSKGVSQYMEKYNNHPKQQSNDVV-K-------------------------     1137
TgGCα        SSCPGLPSTANFSVAA----YPT-------------------------------GGGANS     1296
HsATP8A1     ------------------------------------------------------------     492
HsATP8A2     ------------------------------------------------------------     512

                        S1214

PbGCα        -----YESKT--------------------------------RG-IIRHK---------K     1144
PfGCα        QFSCDYMKNR--------------------------------NDIIFETSTDYYANQDNA     1286
PvGCα        --------MR--------------------------------NELNLKTSMEYDCLEEEE     1157
TgGCα        LFSCSASAFAGPVTQGFPSVTHPAALASDGAAGADEDEEKNVSQLLLPSGTSASSGAPSG     1356
HsATP8A1     ------------------------------------------------------------     492
HsATP8A2     ------------------------------------------------------------     512


PbGCα        KYSHNS--SIF-----------------------DNTK-------------MFSYSINQV     1166
PfGCα        KRKREK--TLF-----------------------DNVSHCGEIQEYRDN-KKYRMKRSQT     1320
PvGCα        HRGV------------------------------------GK---FEDRQTKWITEMSDT     1178
TgGCα        PRGDPQLVSLLGRQGQGHGSLGAPGSGLPTSGCLGGAGGSGARGPMGLHGSRRSLPGSSD     1416
HsATP8A1     ------------------------------------------------------------     492
HsATP8A2     ------------------------------------------------------------     512


PbGCα        FLDGKKARPQ------ASANYY--STKNSLRNENKKNDM--IKKESTFQMFVNKYF----     1212
PfGCα        CSNNRKI-FS------NQRTLY--DYRNMNNNLKNKNSY--LRNKLKSKIFLDKSY----     1365
PvGCα        AKNA--K-KK------NETNLY--NYNTLNN-LKHKN-E--KKNNIKFKFLFNKKF----     1219
TgGCα        CRVPSTARPAPLLGPDAAGRGFGPDYVPSPRPLSPAGPPNASTGSLGEAPKADKPASPEE     1476
HsATP8A1     ------------------------------------------------------------     492
HsATP8A2     ------------------------------------------------------------     512
```

Supp. Fig. S8
