# Supplementary material for: Protein kinase PfPK2 mediated signalling is critical for host erythrocyte invasion by malaria parasite
Source: PLoS Pathog. 2023 Nov 21;19(11):e1011770. doi: 10.1371/journal.ppat.1011770 (PMC10662742; doi:10.1371/journal.ppat.1011770)
Supplement: S1 Table — (PDF) [file ppat.1011770.s009.pdf]

Table S1: PCR Primer used in this study

Purpose	Forward (5'-3')	Reverse (5'-3')
PfPK2 Homology arm: Cloning in pSLI-N-sandwich-loxP vector	1 CACTATAGAATACTCgcggccgcTAAATGGAGAAAAGATAT	2 TATTAACTTCTGCTCgtttaaacAATATTCTAAGACTAAATATACAAATC
PfPK2-loxP: Genotyping 5'-integration	3 GATAAGGGTACATTTATAATTATGATAAAATAGGG	4 AAATCCTCTTCTGATATTAACTTCTGCTCG
PfPK2-loxP: Genotyping 3'-integration	5 Gctatttaggtgacactatagaatactcgc	6 TTTAAATATTCAAATAATTCACCACCCTTT
PfPK2-loxP: Genotyping W.T locus	3 GATAAGGGTACATTTATAATTATGATAAAATAGGG	6 TTTAAATATTCAAATAATTCACCACCCTTT
PfPK2-loxP: Genotyping Episomal	5 Gctatttaggtgacactatagaatactcgc	4 AAATCCTCTTCTGATATTAACTTCTGCTCG
PfPK2-loxP: Excision PCR	3 GATAAGGGTACATTTATAATTATGATAAAATAGGG	7 Aaacgaacattaagctgccatatcc

HA-PfPK2 (KpnI dead mutant):<br>For Cloning in pARL-BSD	8<br>ATATATTATGTGG*A*TACCCACCTTTCC	9<br>GGAAAGGTGGGTA*T*CCACATAATATAT
HA-PfPK2 (WT):<br>Cloning in pARL-BSD Vector<br>(For Complementation)	10<br>CGGggtaccATGTACCCGTACGACGTCCCGGACTACGCTATGGAGAAAAGATATCAGCAATTG	11<br>TCCcctaggCTAATTCTGTGGGGGAGATCTTC

PCR Primer used for recombinant PfPK2 expression

PfPK2 WT (RCO):<br>*Cloning in pET28a vector*	12<br>GTggatccATGGAGAAAAGGTATCAGC	13<br>GCgcggccgcCTAATTTTGTGGTGGACTACG
PfPK2 K140M (RCO):<br>*Template used*<br>*pET28a-PfPK2 WT vector*<br>*(Site-Directed Mutagenesis)*	14<br>AGAGTTGTAGTGA*T*GGAAGTGGATAAG	15<br>CTTATCCACTTCC*A*TCACTACAACTCT
PfPK2 ΔRD (RCO):<br>*Template Used*<br>*pET28a-PfPK2 WT vector*<br>*(Site-Directed Mutagenesis)*	16<br>GATATGCATATGAATACACAAAAT	17<br>ATTTTGTGTATTCATATGCATATC

PfPK2 ΔC (RCO): Cloning in pET28a vector	12 GTggatccATGGAGAAAAGGTATCAGC	18 AGgcggccgcGTCATTGTAATTGTTTTTGTG
PfPK2 ΔRD+ΔC (RCO): Cloning in pET28a vector	12 GTggatccATGGAGAAAAGGTATCAGC	19 AAGGAAAAAAgcggccgcTTAGGTTAATTTTGGTAAAAACCAC
